# Intraintestinal Analysis of the Functional Activity of Microbiomes and Its Application to the Common Marmoset Intestine

**DOI:** 10.1128/msystems.00520-22

**Published:** 2022-08-25

**Authors:** Mika Uehara, Takashi Inoue, Minori Kominato, Sumitaka Hase, Erika Sasaki, Atsushi Toyoda, Yasubumi Sakakibara

**Affiliations:** a Department of Biosciences and Informatics, Keio University, Yokohama, Kanagawa, Japan; b Department of Marmoset Biology and Medicine, Central Institute for Experimental Animals, Kawasaki, Kanagawa, Japan; c Department of Genomics and Evolutionary Biology, National Institute of Genetics, Mishima, Shizuoka, Japan; d Laboratory for Marmoset Neural Architecture, RIKEN Center for Brain Science, Saitama, Japan; University of Connecticut

**Keywords:** biogeography, bioinformatics, common marmoset, intestine, metagenome, metatranscriptome, primate

## Abstract

The intestinal microbiome is closely related to host health, and metatranscriptomic analysis can be used to assess the functional activity of microbiomes by quantifying microbial gene expression levels, helping elucidate the interactions between the microbiome and the environment. However, the functional changes in the microbiome along the host intestinal tract remain unknown, and previous analytical methods have limitations, such as potentially overlooking unknown genes due to dependence on existing databases. The objective of this study is to develop a computational pipeline combined with next-generation sequencing for spatial covariation analysis of the functional activity of microbiomes at multiple intestinal sites (biogeographic locations) within the same individual. This method reconstructs a reference metagenomic sequence across multiple intestinal sites and integrates the metagenome and metatranscriptome, allowing the gene expression levels of the microbiome, including unknown bacterial genes, to be compared among multiple sites. When this method was applied to metatranscriptomic analysis in the intestinal tract of common marmosets, a New World monkey, the reconstructed metagenome covered most of the expressed genes and revealed that the differences in microbial gene expression among the cecum, transverse colon, and feces were more dynamic and sensitive to environmental shifts than the abundances of the genes. In addition, metatranscriptomic profiling at three intestinal sites of the same individual enabled covariation analysis incorporating spatial relevance, accurately predicting the function of a total of 10,856 unknown genes. Our findings demonstrate that our proposed analytical method captures functional changes in microbiomes at the gene resolution level.

**IMPORTANCE** We developed an analysis method that integrates metagenomes and metatranscriptomes from multiple intestinal sites to elucidate how microbial function varies along the intestinal tract. This method enables spatial covariation analysis of the functional activity of microbiomes and accurate identification of gene expression changes among intestinal sites, including changes in the expression of unknown bacterial genes. Moreover, we applied this method to the investigation of the common marmoset intestine, which is anatomically and pharmacologically similar to that of humans. Our findings indicate the expression pattern of the microbiome varies in response to changes in the internal environment along the intestinal tract, and this microbial change may affect the intestinal environment.

## INTRODUCTION

The intestinal tract regulates highly complex physiological processes while interacting with a dense and diverse microbial population. The large intestine, in particular, has a high-density microbiome, which is important in host-microbiome interactions (1). Most studies use fecal samples based on the assumption that feces reflect the condition of the microbiome inside the intestinal tract (2–5). Because the function of the intestinal tract varies from site to site and there are differences in the physicochemical environment, such as in nutrient, oxygen, and pH levels, the microbiome may differ in its response to changes in the environment (6, 7). Indeed, due to these environmental shifts, some studies have reported that the composition of the microbiome varies depending on the intestinal site (biogeographic locations) in model animals (8–12). However, it is still unclear how microbial function varies along the intestinal tract, as these studies have shown only differences in the microbial members in the intestinal tract, and metatranscriptomic analysis has not yet been conducted.

Moreover, to study the interrelationship between humans and microbiomes, an animal model that is highly anatomically and pharmacologically similar to humans is more appropriate. The common marmoset is a small New World primate that is considered a useful model in preclinical studies due to its common physiological and anatomical characteristics with those of humans (13). In addition, the common marmoset is the only nonhuman primate in which germfree conditions have been successfully produced, and it has the potential to expand the scope of intestinal microbiome studies (14).

In the past few decades, many sequence-based analyses have attempted to elucidate the relationships between microbiomes and environments such as the ocean, soil, and digestive tract. These studies have traditionally focused on profiling membership through amplicon sequencing of the 16S rRNA gene. Recently, whole-metagenomic sequencing methods, which enable comprehensive capture of microbial genomes to reconstruct database-independent metagenome sequences and reveal potential microbial genes and community taxonomic abundance profiles, have been more widely used due to advances in sequencing throughput and analytical methods. For instance, in a large-scale metagenomic analysis spanning human body parts—the oral cavity, skin, feces, and vagina—154,723 microbial genomes were reconstructed, 77% of which were unknown genomes not found in public repositories (2). Additionally, a study on the cow rumen microbiome reported 913 microbial genomes, and these reconstructed genomes improved the metagenomic read classification 7-fold (15). Other studies have shown microbial genes detected in reconstructed metagenomic sequences play an important role in the pathology of rheumatoid arthritis (3). Although these metagenomic studies have provided many insights into a wide variety of microbiomes by finding new bacterial genomes and potential genes and have emphasized the importance of reconstructing bacterial genomes, these approaches only show the presence of microbiome members and their genes and cannot indicate whether they are active members of the microbiome or how the bacteria actually interact with the environment. As a way to solve these problems, metatranscriptomic analysis of transcripts within a microbiome can be used to obtain deeper insight into how bacterial communities respond to environmental conditions. A study that included both metatranscriptomic and metagenomic analyses in patients with inflammatory bowel disease (IBD) highlighted the metabolic pathways characteristic of the disease and revealed whether metagenomically abundant bacteria were inactive or dormant in the intestine (4). In a human fecal microbiome study with both metagenomic and metatranscriptomic analyses, the metatranscriptome was found to be more dynamic than the metagenome, and there was a discrepancy between bacterial abundance and transcriptional activity (5). As such, finding microbial gene expression signatures can be crucial to understanding the mechanisms underlying microbe-environment interactions.

Metatranscriptomic analysis utilizes two main approaches to quantify bacterial transcripts, each with its own drawbacks. The first is the read-based approach used in pipelines such as HUMAnN2 (16) and SAMSA2 (17), which assess the activity of each protein family and pathway by aligning reads derived from metatranscriptomic library preparations with protein databases such as RefSeq (18) and pathway databases such as KEGG (19) and MetaCyc (20), respectively. This method is simple and often used but may miss many previously unknown genes that are not annotated in the databases.

The second approach involves metatranscriptomic analysis based on *de novo* assembly of metagenomic data. Gene expression is quantified by aligning RNA reads with the predicted genes for contigs obtained by assembling corresponding metagenomic DNA reads, which requires simultaneous sampling of the metagenome and metatranscriptome from the same sample. This approach is powerful enough to discover and focus on unknown genes and was, therefore, adopted in the present study. When applied to the analysis of the microbiome in multiple environments, the challenge with this approach is to identify the same gene across samples, because the assembled genomic sequence varies from base to base depending on the sample.

In the present study, we aimed to clarify the changes in microbial abundance and gene expression caused by environmental gradients among the cecum, transverse colon, and feces. To accurately perform this investigation, it is necessary to overcome the discrepancy between the microbes existing in the environment and those registered in databases such as the COG and KEGG Orthology (KO) databases (19, 21). We developed an integrated metagenomic and metatranscriptomic method for analyzing the functional changes in microbiomes across multiple intestinal sites and predicting the functions of unknown genes and then applied this method to the investigation of the common marmoset intestine. Our method not only reconstructed the metagenome more accurately than conventional methods but also overcame the bottleneck of identifying the corresponding same gene among multiple samples. By applying this approach, we predicted the functions of 10,856 unknown genes by spatial covariation analysis.

## RESULTS

### Overview of the proposed analytical method that integrates the metagenome and metatranscriptome to analyze the functional activity of microbiomes among intestinal sites.

**FIG 1:**
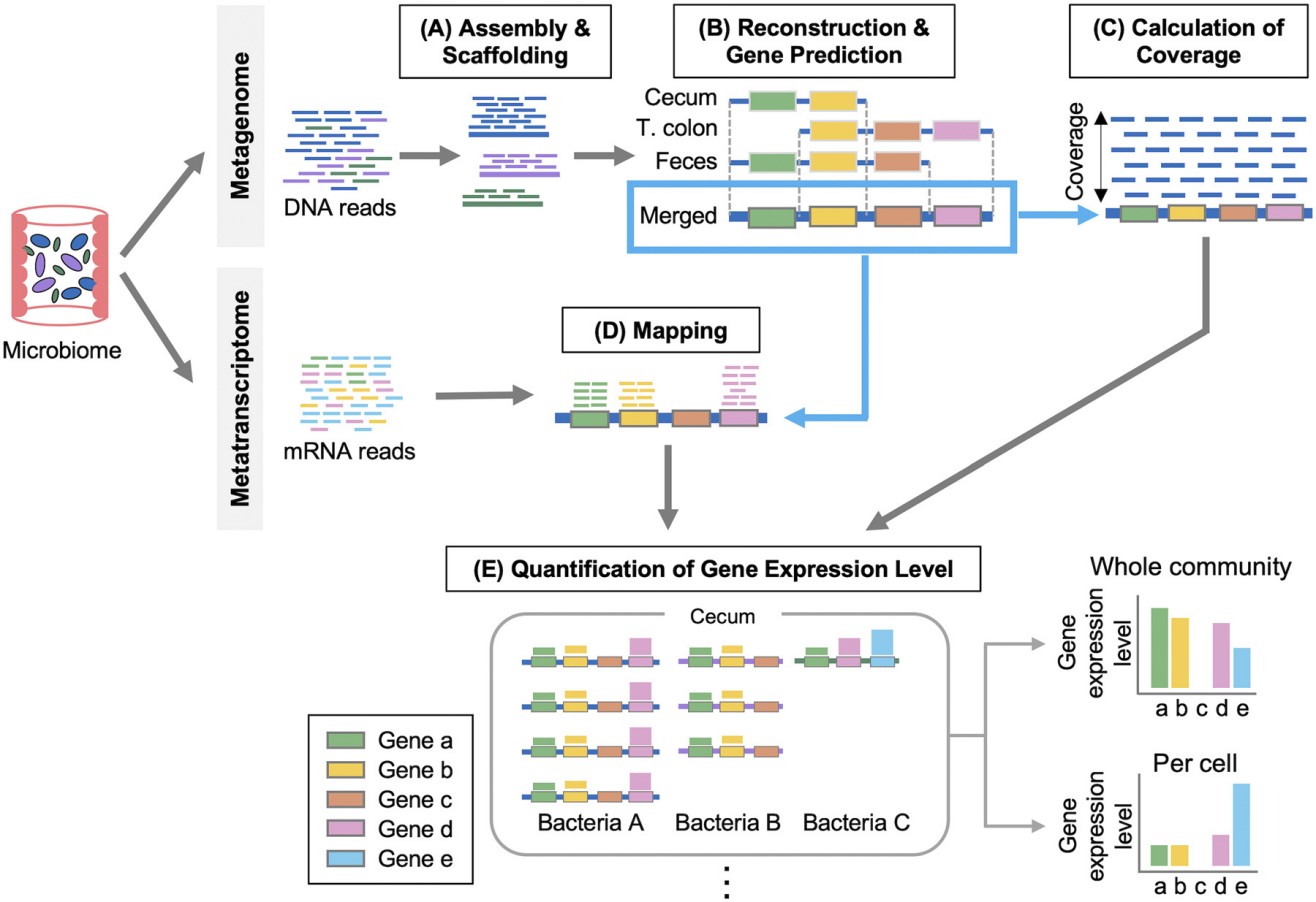
Overview of the proposed analytical method. This method integrates the metagenome and metatranscriptome to compare the functional activity of microbiomes among intestinal sites as follows. Samples for the metagenomic and the metatranscriptomic analyses are taken simultaneously. (A) Assembly with DNA reads generates contigs and scaffolds at each site. (B) The common reference metagenomes are reconstructed by merging scaffolds across all sites. Gene-coding regions are predicted in the reconstructed metagenome. (T. colon represents the transverse colon). (C) DNA reads are mapped to the reconstructed metagenome to calculate relative abundance. (D) mRNA reads are aligned to the reconstructed metagenome, and mapped reads are quantified for each gene. (E) Gene expression levels are calculated for the whole community. Gene expression levels per cell are calculated by normalizing to gene abundance.

After assembly and scaffolding of the metagenomic reads, the proposed analytical method adopted a strategy to reconstruct the common reference metagenomes in multiple sites, including those of unknown bacteria, by merging the scaffolded contigs among samples; accordingly, the expression levels of all bacterial genes could be quantified by integrating this reconstructed reference metagenome with metatranscriptomic data. An overview of the proposed analytical method is illustrated in Fig. 1. Using this method, we compared the microbial gene expression levels among three sites—the cecum, transverse colon, and feces. These sites were selected as locations equivalent to the proximal, middle, and distal positions of the colon, where most bacteria are located (22). In addition, we compared the corresponding microbial compositions among humans, mice, rats and marmosets to evaluate the suitability of the common marmoset as a preclinical animal model for microbiome studies.

### Metagenome reconstruction improves assembly contiguity, transcript mapping rate, and identification of the same genes among sites.

**FIG 2:**
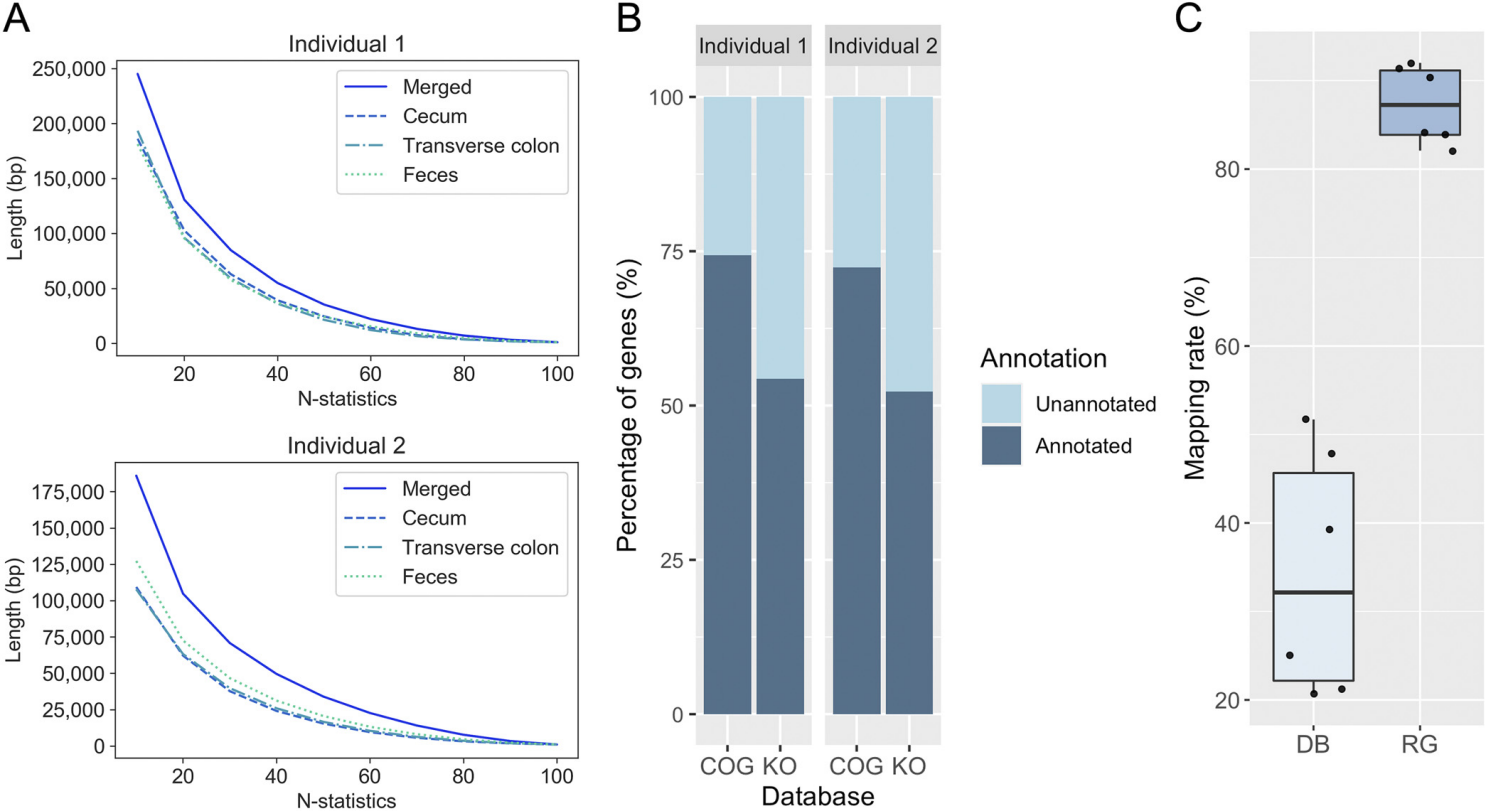
Reconstruction of a metagenome by merging improved the assembly contiguity, gene detection and read mapping rate. (A) The plots of N-statistics to measure the assembly contiguity reconstructed from three intestinal sites and of the merged metagenome in individuals 1 and 2. We computed the N-statistics from N10 to N100 at 10 intervals, which is an extension of the N50 measure to evaluate assembly contiguity. (B) Percentage of functionally annotated genes in the reconstructed genomes. Approximately 63,331 and 88,575 genes were not present in the COG database, and 112,790 and 152,845 genes were not present in the KO database. (C) Mapping rate of microbial mRNA reads to the database and the reference metagenome (DB, database; RG, reconstructed reference metagenome). This boxplot represents the mapping rates of mRNA reads from the cecum, transverse colon, and feces in individuals 1 and 2.

Reference metagenomes with total lengths of 306 Mb and 395 Mb, consisting of 32,244 and 39,905 scaffolds, respectively, were reconstructed by merging metagenomes from three intestinal sites for individuals 1 and 2, respectively. We compared the scaffold lengths before and after merging scaffolds from three sites by a generalized N-statistic score, which is an extension of N50. Scores from N10 to N100 were plotted at 10 intervals, as shown in Fig. 2A. The score for the genome assembly of each intestinal site fell well below that of the merged one. This implied that merging improved the assembly contiguity, indicating the assembled scaffolds from the three sites complemented each other to reconstruct a longer genome (Fig. S1; Text S1). Next, 246,980 and 320,613 genes were detected in the reconstructed metagenomes for individuals 1 and 2, respectively. Of the genes detected in individuals 1 and 2, 63,331 and 88,575 (26% and 28%) genes were not present in the COG database, and 112,790 and 152,845 (46% and 48%) genes were not present in the KEGG database (Fig. 2B; Table S1). Thus, a large number of novel genes not included in the public database were detected in the reconstructed metagenomes.

10.1128/msystems.00520-22.1 FIG S1 Visualization of scaffold alignment to the merged genome (MG_34075) by IGV, related to Fig. 2A. The grey segment represents the scaffold in each site, and the purple markers represents an insertion within the scaffold. The scaffolds across the sites complement each other to reconstruct a large genome (Text S1). Download FIG S1, PDF file, 0.02 MB. Copyright © 2022 Uehara et al. 2022 Uehara et al. https://creativecommons.org/licenses/by/4.0/ This content is distributed under the terms of the Creative Commons Attribution 4.0 International license.

10.1128/msystems.00520-22.4 TABLE S1 Number of annotated genes. Download Table S1, DOCX file, 0.02 MB. Copyright © 2022 Uehara et al. 2022 Uehara et al. https://creativecommons.org/licenses/by/4.0/ This content is distributed under the terms of the Creative Commons Attribution 4.0 International license.

10.1128/msystems.00520-22.10 TEXT S1 **Supplementary Notes.** Supplementary information on the methods, consisting of: Section 1. Visualization of scaffold alignment to the merged genome by IGV; Section 2. Percentage of genes that match in scaffolds of all three sites and the reconstructed scaffold; Section 3. Functional annotation for unknown genes by covariation analysis; Section 4. Validation of covariation analysis by sequence similarity of linked gene clusters; Section 5. Assessment of reconstructed metagenomics; Section 6. Removal of contaminated sequence; Section 7. Computation method of gene expression level per cell; Section 8. Parameter determination and evaluation of the integrated analytical method; Section 9. Information of the data on animals other than marmosets used for 16S rRNA analysis; Section 10. Evaluation metrics for comparing the metagenomic reconstruction methods. Download Text S1, PDF file, 0.1 MB. Copyright © 2022 Uehara et al. 2022 Uehara et al. https://creativecommons.org/licenses/by/4.0/ This content is distributed under the terms of the Creative Commons Attribution 4.0 International license.

To quantify the gene expression levels, we first mapped the mRNA reads to all complete bacterial, archaeal, and viral genomes in the RefSeq database (18). Only 21% to 52% of the mRNA reads could be assigned to the known genomes (Fig. 2C). This result confirmed that information to understand microbiome activity was limited if relying solely on genomes registered in public databases. We therefore mapped the mRNA reads to the reference metagenomes reconstructed in this study. The mapping rate to the reconstructed metagenomes increased to 82% to 92% (Fig. 2C). The reconstructed metagenomes covered most of the expressed genes (Table S2) and allowed us to map 2 to 4 times more reads in comparison to the public databases. These results underscore the importance of database-independent analytical methods, especially in metatranscriptomic analysis, to quantify microbial gene expression levels.

10.1128/msystems.00520-22.5 TABLE S2 Number of expressed genes at each site. Download Table S2, DOCX file, 0.02 MB. Copyright © 2022 Uehara et al. 2022 Uehara et al. https://creativecommons.org/licenses/by/4.0/ This content is distributed under the terms of the Creative Commons Attribution 4.0 International license.

In addition, we verified that the gene annotations were retained before and after merging by examining the percentage of genes common to the three sites that matched the corresponding genes in the reconstructed metagenome. We found that 96.9% and 96.6% of the genes common to the three sites were identical to those in the merged metagenome in individuals 1 and 2, respectively (allowing for a 3-base mismatch; Text S1; Table S3). The reference metagenome reconstructed by merging thus achieved high accuracy in identifying the same genes among three intestinal sites.

10.1128/msystems.00520-22.6 TABLE S3 Percentage of genes that match in scaffolds of all three sites and reconstructed scaffolds. Download Table S3, DOCX file, 0.02 MB. Copyright © 2022 Uehara et al. 2022 Uehara et al. https://creativecommons.org/licenses/by/4.0/ This content is distributed under the terms of the Creative Commons Attribution 4.0 International license.

### Functional annotation of unknown genes with metatranscriptomic profiles.

**FIG 3:**
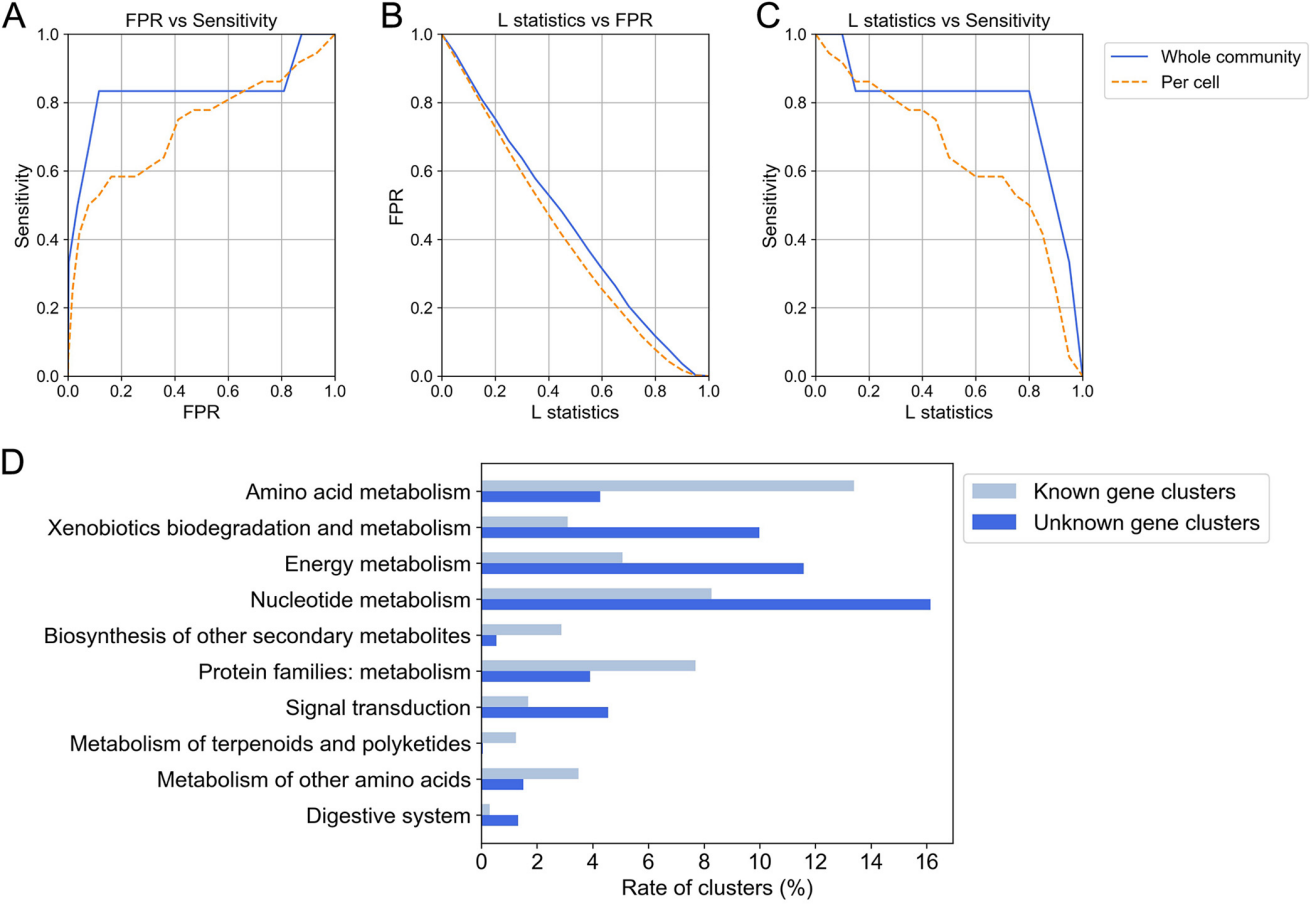
Rationale for the covariation analysis and the resulting molecular functions associated with unknown genes. Covariation analysis incorporating bivariate spatial relevance was performed to associate unknown genes with molecular functions. Evaluation of the covariation analysis results using the gene expression profile of the whole community and per cell: (A) receiver operating characteristic (ROC) curves of false positive rate (FPR) versus sensitivity, (B) FPR curves and (C) sensitivity curves along the L statistic value to associate known gene cluster pairs. True positives were defined as pairs of covariant genes with a common KEGG reaction definition. (D) The functions of unknown gene clusters associated by covariation analysis using the gene expression profile of the whole community and the functions of known gene clusters. Only functions enriched in either unknown or known gene clusters are shown (Fisher's exact test with *P* value < 0.01 adjusted by the Benjamini-Hochberg method; Text S1; Table 9 posted at https://doi.org/10.5281/zenodo.6787048). The L statistic value that ensured an FPR < 0.05 in the benchmark was used as the threshold (Text S1).

To address unknown genes that were not annotated by the databases, we generated a gene catalogue from the reconstructed metagenomic sequences by grouping the genes into clusters and performing a covariation analysis. Of the unknown genes detected in two individuals, 50,509 expressed genes were grouped into 24,725 gene clusters based on protein sequence similarity (Table 7 posted https://doi.org/10.5281/zenodo.6787048). In addition, we performed a covariation analysis that estimated the functions of the unknown gene clusters (23), incorporating bivariate spatial relevance (24) between multiple intestinal sites. We first evaluated the rationale of this spatial covariation analysis in which pairs of genes with similar expression profiles were associated with a common metabolic process (Text S1; Table 8 posted at https://doi.org/10.5281/zenodo.6787048). As a result of benchmarking the covariation analysis using the gene expression level at the whole-community and per-cell levels, the areas under the curves (AUCs) of false positive rate (FPR) versus sensitivity along the L statistic value were 0.830 and 0.729, respectively (Fig. 3A), indicating this covariation analysis method is sufficiently effective. The covariation analysis was then applied to the unknown gene clusters with a threshold 0.885 of the L statistic value that ensured an FPR less than 0.05 and a sensitivity greater than 0.66 (Fig. 3B and C; Text S1) for the whole community. The results (Fig. 3D; Table 9 posted at https://doi.org/10.5281/zenodo.6787048) showed the functions of 10,856 unknown genes were predicted and that many of the unknown genes could be involved in xenobiotic biodegradation, energy metabolism, nucleotide metabolism, signal transduction, and the digestive system, which also suggests that current database-dependent analytical methods may underestimate these functions in our data. Thus, covariation analysis incorporating bivariate spatial relevance, combined with metatranscriptomic analysis, provided an accurate functional interpretation of unknown genes in the reconstructed metagenome.

### Spatial variance in microbial gene expression at the whole-community and individual-cell levels.

The functional activity of the microbiome in the cecum, transverse colon, and feces was assessed using both the whole-community and per-cell gene expression levels (Fig. S2). The whole-community gene expression level indicates the functional profile of the entire microbiome but is affected by the abundance of bacteria; on the other hand, the gene expression level per cell indicates the gene activity for each bacterium, even for minority bacteria.

10.1128/msystems.00520-22.2 FIG S2 Pearson correlation coefficient (r) and *P* value (p) between the gene expression level at the whole community and that per cell. The x axis indicates the gene expression level per cell, and the y axis indicates the gene expression level in the whole community. The value converted to log_2_ is used for the expression level. The data points represent expressed genes detected on the reconstructed metagenomes. Download FIG S2, PDF file, 0.6 MB. Copyright © 2022 Uehara et al. 2022 Uehara et al. https://creativecommons.org/licenses/by/4.0/ This content is distributed under the terms of the Creative Commons Attribution 4.0 International license.

**FIG 4:**
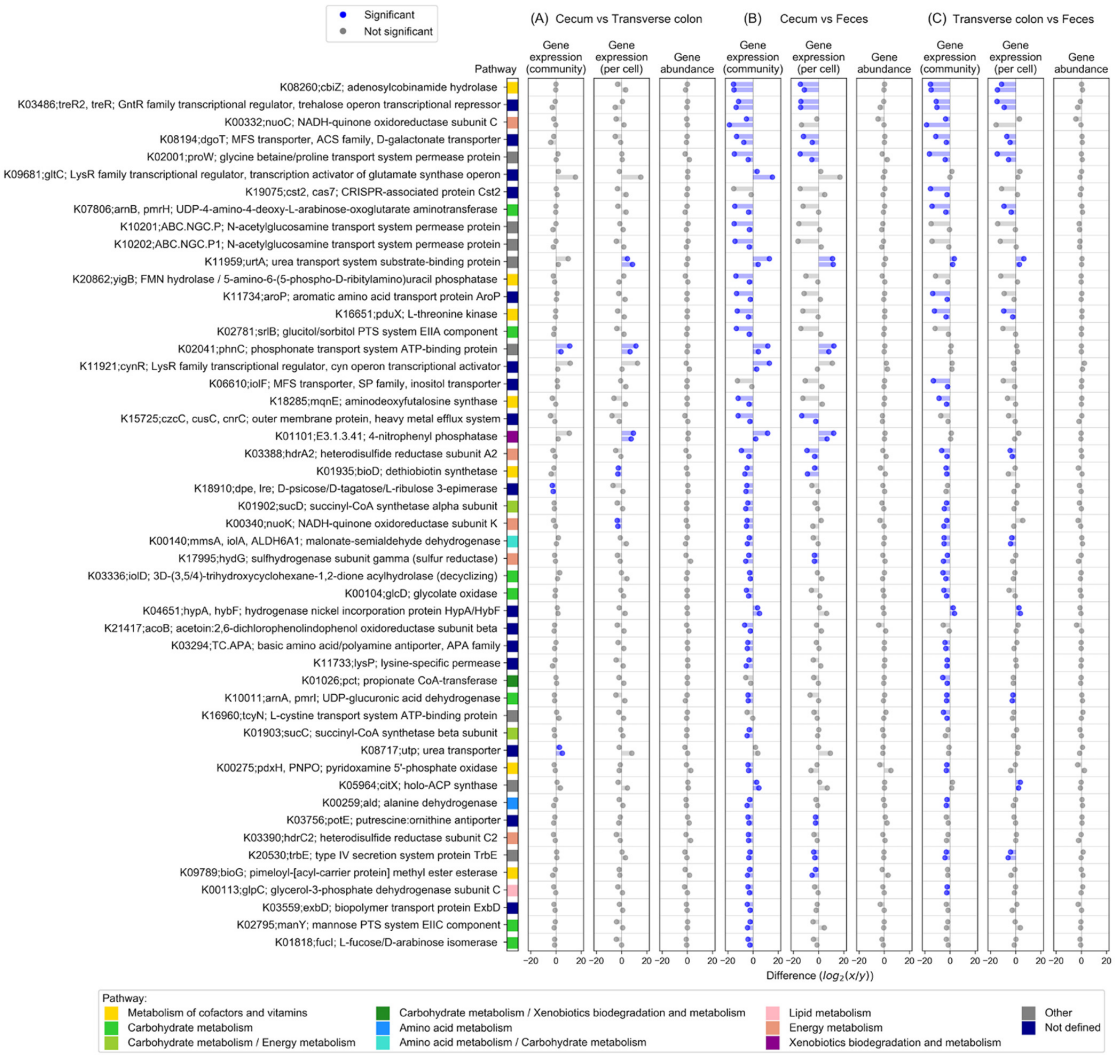
Significant KEGG Orthology of differentially expressed genes between the cecum, transverse colon, and feces. The top 50 KOs with the highest differential gene expression between either pair of sites at the whole-community level are shown, along with gene expression levels per cell and gene abundance. (A) Differential gene expression between the cecum and transverse colon; (B) differential gene expression between the cecum and feces; and (C) differential gene expression between the transverse colon and feces. The difference in gene abundance/expression levels between the whole community and per cell is displayed using log_2_-transformed values. For each KO, the upper bar represents individual 1, and the lower bar represents individual 2. The difference was considered and is denoted “significant” if the difference changed in the same direction by more than 2-fold in both individuals.

We identified the biochemical functions whose expression levels varied significantly among the intestinal sites. The top 50 KOs with the highest expression differences between either pair of sites at the whole-community level are listed in Fig. 4, along with gene expression levels per cell and gene abundance. The KOs K02041 (phosphonate transport system ATP-binding protein), K18910 (d-psicose/d-tagatose/l-ribulose 3-epimerase), and K08717 (urea transporter) were differentially expressed between the cecum and transverse colon (Fig. 4A). The differentially expressed KOs between the cecum and feces were 45 of the top 50 KOs, including K08260 (adenosylcobinamide hydrolase), K03486 (GntR family transcriptional regulator, trehalose operon transcriptional repressor), and K00332 (NADH-quinone oxidoreductase subunit C) (Fig. 4B). K19075 (CRISPR-associated protein Cst2) and 29 more KOs were differentially expressed between the transverse colon and feces (Fig. 4C). Here, we focused on genes involved in well-studied metabolic processes. The genes that were more highly expressed in the whole community in the cecum and transverse colon than in the feces were genes involved in the biosynthesis of vitamin B12 (*cbiZ* and *pduX*), vitamin K_2_ (*mqnE*), vitamin B_7_ (*bioD*), and vitamin B6 (*pdxH*) and antibiotic resistance genes (*arnA* and *arnB*) (Fig. 4B and C). The gene *cbiZ*, which salvages cobinamide (Cbi), a precursor of AdoCbl, originated in archaea and was acquired by several bacterial strains via horizontal gene transfer (25). This gene is required for bacterial growth on acetate (26). The detection of *pduX* as a differentially expressed gene along with *cbiZ* is consistent with a previous study showing that *pduX* is required for the *cbiZ*-mediated pathway (27). Two genes, *arnA* and *arnB*, are known to confer resistance to antibiotics by modifying the outer membrane with lipopolysaccharide. This modification is regulated by the PmrA/PmrB two-component regulatory system, which is switched on by low pH (28).

**FIG 5:**
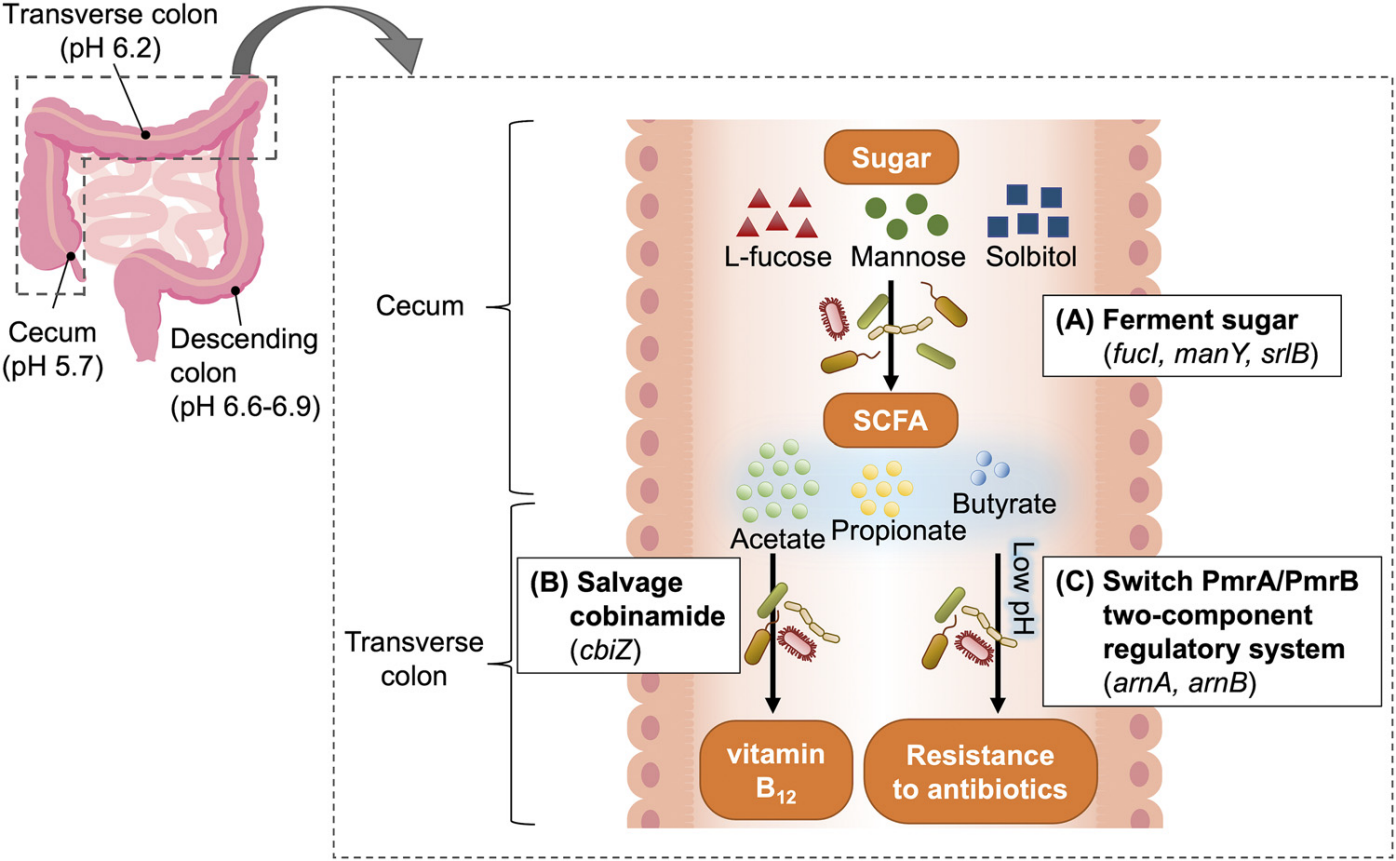
Functional activities in the microbiome along the host intestinal tract. In relation to Fig. 4, the functional shifts of the microbiome along the intestinal tract estimated from the differentially expressed genes between sites were as follows: (A) Sugars that are not absorbed in the small intestine are fermented by the cecal microbiome to produce SCFAs (29, 30). As SCFAs are absorbed in the large intestine, the concentration of SCFAs gradually decreases from the cecum to the descending colon (31). (B) The growth of bacteria under acetate (26), which is abundant in SCFAs (32), requires *cbiZ* in the vitamin B12 biosynthetic pathway, and the production of SCFAs makes this gene more active in the cecum and transverse colon than in feces. (C) Similarly, a decrease in pH with increasing SCFA concentration switches on the antibiotic resistance genes *arnA* and *arnB* (28). The pH value at each intestinal site displayed in the left figure was taken from previous studies (64).

**FIG 6:**
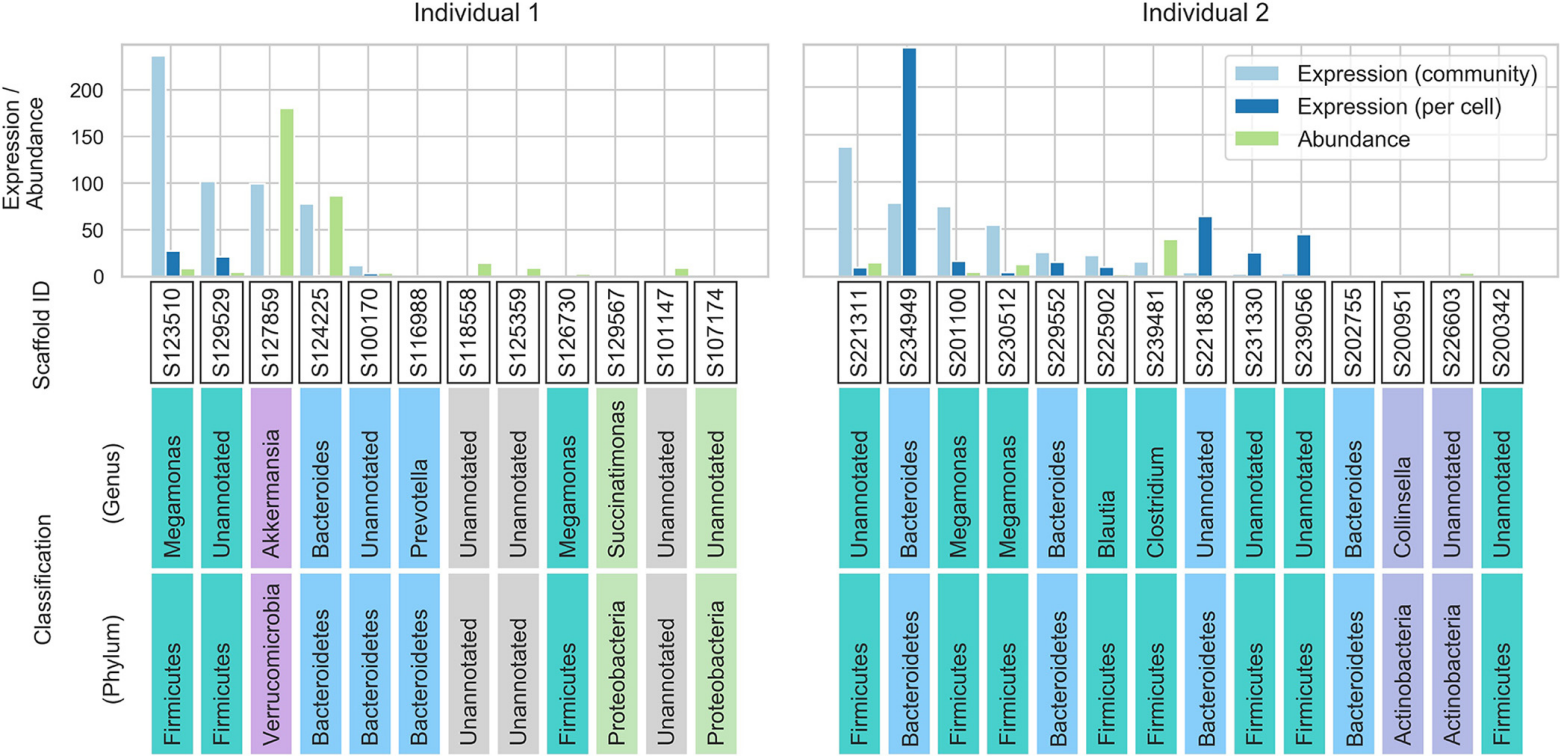
Thirty scaffolds encoding the L-fucose isomerase gene (*fucI*), each representing one bacterial species. The bar plot shows the relative gene abundance and expression of *fucI* at the whole-community and per-cell levels from 30 scaffolds in individuals 1 and 2. Each scaffold ID and its taxonomic classification are shown at the bottom and colored by phylum-level classification.

These differentially expressed genes are related to sugar utilization in the intestinal tract (Fig. 5). The genes that were differentially expressed at the whole-community level between the cecum and feces were the genes involved in the utilization of sorbitol (*srlB*), mannose (*manY*), and l-fucose (*fucI*) (Fig. 5A). This result likely reflects the utilization of sugars that were not absorbed in the small intestine by the microbiome (29). Fermentation of these sugars by the cecal microbiome produces short-chain fatty acids (SCFAs) (30), increasing the concentration of SCFAs in the colon; however, the level of SCFAs decreases in feces due to their absorption in the colon (31). Acetic acid accounts for approximately 60% of SCFAs in the colon (32) and, therefore, this change in the concentration of SCFAs along the colon explains the changes in the expression of *cbiZ* (Fig. 5B), which is essential for bacterial growth on acetate (26). Similarly, the decrease in the concentration of SCFAs from the cecum to the descending colon was accompanied by an increase in pH, which is consistent with the changes in the expression of the antibiotic resistance genes *arnA* and *arnB*, which are activated at low pH (28) (Fig. 5C). Thus, many of these genes that were differentially expressed between intestinal sites are associated with the SCFAs produced by microbial sugar metabolism. As these typical genes are obviously encoded in multiple bacterial species, we selected the l-fucose metabolic gene (*fucI*) and identified the bacteria that exhibited differential expression of this gene. In the reconstructed reference metagenome, 30 loci encoding *fucI* were detected, each representing one bacterial species (Fig. 6). This analysis showed many bacteria (scaffolds) belonging to the Firmicutes phylum contributed to the expression level of the *fucI* gene at the whole-community level and scaffold ID S123510, belonging to the *Megamonas* genus, was an important contributor in individual 1. On the other hand, scaffold ID S127859, belonging to the *Akkermansia* genus, a well-known SCFA-producing bacterial genus (33), showed the greatest abundance of *fucI* in individual 1, although the expression level per cell was low. This finding demonstrates the power of our method of integrating metagenomes and metatranscriptomes to enable analysis at the gene level.

### Comparison of microbiomes among animal models by 16S rRNA gene sequencing.

**FIG 7:**
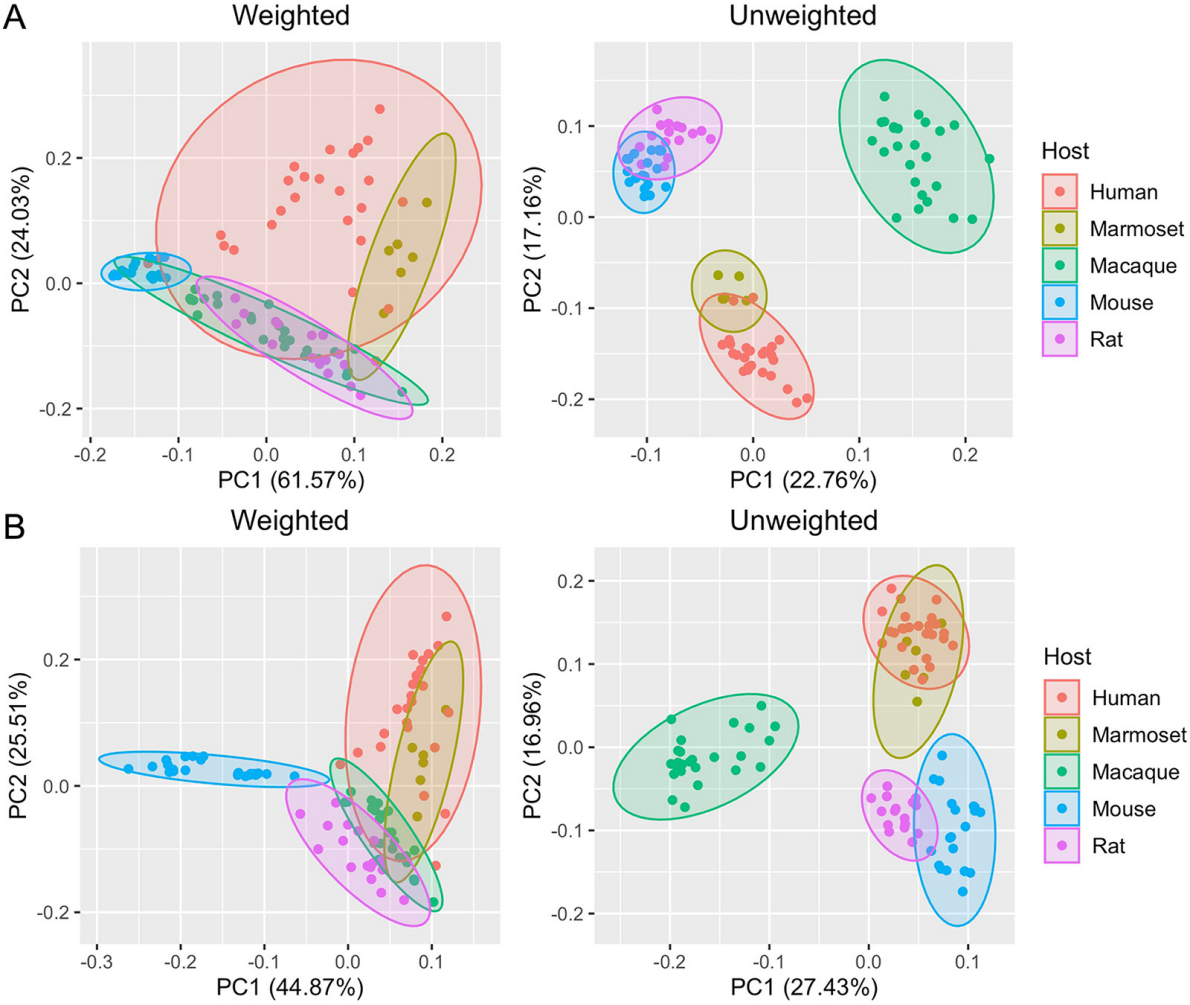
Comparison of the fecal microbiomes of marmosets, mice, rats, macaques, and humans. OTU-based unweighted and weighted PCA (A) at the genus level and (B) at the family level. The 16S rRNA gene sequence data for fecal samples from six marmosets were obtained in this study. The 16S rRNA gene sequence data for fecal samples from humans, macaques, rats, and mice were obtained from a previous study (34).

To identify similarities and differences between the common marmoset microbiome and those from humans and other major model animals (macaques, mice, and rats), 16S rRNA amplicon sequencing of marmoset fecal samples was conducted. The 16S rRNA gene sequence data for fecal samples from humans, macaques, rats, and mice were obtained from a previous study (34). Operational taxonomic unit (OTU) analysis of microbiome similarity was performed quantitatively (weighted) and qualitatively (unweighted) at the genus and family levels. The results of principal-component analysis (PCA) of the OTU profiling data are shown in Fig. 7. In contrast to the weighted analysis, the unweighted analysis more clearly isolated clusters of species. The marmoset clusters overlapped with human clusters in both weighted and unweighted analyses, revealing the marmoset and human microbiomes were most similar. Mouse and rat clusters were located near each other in the unweighted analysis. The analysis at the family level showed the *Muribaculaceae* family accounted for approximately half of the microbiome of mice and was also detected in rat and macaque individuals (Fig. S3). In contrast, most humans and marmosets did not retain *Muribaculaceae* (Fig. S3). Despite all three groups being primates, the macaque microbiome did not resemble the marmoset or human microbiome in the unweighted profile, and no specific common bacteria were detected between macaques and humans or between macaques and marmosets. On the other hand, characteristic bacteria were found in the comparison between marmosets and humans. The *Bacteroidaceae* family and *Bacteroides* genus are major members in marmosets and humans. *Bacteroides*, which inhabits healthy human intestines, has been reported to have a reduced abundance in IBD patients and is attracting attention as a probiotic (35). The *Bifidobacteriaceae* family, *Bifidobacterium* genus, *Coriobacteriaceae* family, and *Collinsella* genus were also present in most marmoset and human individuals but were not detected in many individuals of other animal model species. *Bifidobacterium* is known to be significantly depleted in individuals with colorectal cancer, IBD, irritable bowel syndrome, and obesity and has been reported to enhance the effectiveness of cancer immunotherapy (36, 37). *Collinsella* is a proinflammatory genus involved in rheumatoid arthritis and nonalcoholic steatohepatitis and has potential applications as a disease biomarker (38, 39). In brief, it was found that the marmoset and human fecal microbiomes are significantly similar and share many bacteria involved in a variety of human diseases.

10.1128/msystems.00520-22.3 FIG S3 Relative abundance of bacterial families in marmosets, humans, macaques, rats, and mice, related to Fig. 7. The 16S rRNA gene sequence data for fecal samples from six marmosets were sequenced in this study. The 16S rRNA gene sequence data for fecal samples from humans, macaque monkeys, rats, and mice were obtained from a previous study (34; https://doi.org/10.3389/fmicb.2018.02897). Download FIG S3, PDF file, 0.9 MB. Copyright © 2022 Uehara et al. 2022 Uehara et al. https://creativecommons.org/licenses/by/4.0/ This content is distributed under the terms of the Creative Commons Attribution 4.0 International license.

## DISCUSSION

The method proposed in this study was used to reconstruct the common reference metagenome by merging scaffolds assembled from metagenomic read data from three different intestinal sites; using this approach, it was possible to identify the corresponding genes among three intestinal sites with high accuracy. Here, we evaluated the nonchimeric rate of the reconstructed genomes using a benchmarking data set that collected only DNA reads assigned to known bacterial species. The nonchimeric rate is defined by the percentage of the genome length assembled solely with DNA reads from a single species. The nonchimeric rates were 92.8% and 94.7% for individuals 1 and 2, respectively; this revealed that most genomes were completely reconstructed as a single species within the metagenome (Text S1).

In addition, we compared the proposed method for merging metagenomic contigs among the multiple sites with six other methods, including a coassembly method that assembles all the reads from multiple sites together. As a result of evaluation by the composite performance metric (CPM) (40) reflecting both contiguity and accuracy for an assembly, the proposed method achieved the highest score (Table S4 and S5). Furthermore, our method not only reconstructed the metagenome more accurately than conventional methods (Table S6) but also overcame the bottleneck of identifying the corresponding same gene among multiple samples. By this approach, we predicted the function of 10,856 unknown genes by spatial covariation analysis (Fig. 3).

10.1128/msystems.00520-22.7 TABLE S4 N50, composite performance metric (CPM), chimera index (CI), max alignment length (MAL), and total alignment length (TAL) of the metagenome reconstructed by each method. Download Table S4, DOCX file, 0.02 MB. Copyright © 2022 Uehara et al. 2022 Uehara et al. https://creativecommons.org/licenses/by/4.0/ This content is distributed under the terms of the Creative Commons Attribution 4.0 International license.

10.1128/msystems.00520-22.8 TABLE S5 Average of CI and N50 of two individuals in Table S4 (1 to 6) and those of the metagenome merging the two individuals (7). Download Table S5, DOCX file, 0.02 MB Copyright © 2022 Uehara et al. 2022 Uehara et al. https://creativecommons.org/licenses/by/4.0/ This content is distributed under the terms of the Creative Commons Attribution 4.0 International license.

10.1128/msystems.00520-22.9 TABLE S6 CI and N50 of metagenome of each site and merged metagenome among three sites. Download Table S6, DOCX file, 0.02 MB. Copyright © 2022 Uehara et al. 2022 Uehara et al. https://creativecommons.org/licenses/by/4.0/ This content is distributed under the terms of the Creative Commons Attribution 4.0 International license.

The changes in gene expression among the cecum, transverse colon, and feces were shown to be more dynamic than the changes in microbiome abundance, which was consistent with the results of a previous study (5, 41). This result is also consistent with a previous study based on 16S rRNA gene analysis (8), which concluded that the microbiome composition in feces reflects that in the large intestine in terms of abundance. However, we found the gene expression levels of the microbiome varied among the cecum, transverse colon, and feces. For example, we found genes related to carbohydrates were activated in the cecum compared with the feces, and coenzyme metabolism genes and antibacterial resistance genes were more highly expressed in both the cecum and transverse colon than in the feces, but these gene abundances did not vary significantly. As the differential expression of these genes was considered to be influenced by the concentration of SCFAs derived from carbohydrates by the microbiome, we focused on the *fucI* gene, which is involved in carbohydrate metabolism. The reconstructed reference metagenome identified 30 bacteria (scaffolds) encoding *fucI*, and the *Megamonas* genus contributed the most to the expression of *fucI* at the whole-community level, despite the highest abundance of *fucI* being observed in *Akkermansia*. SCFAs are involved in host lipid metabolism (42), and *Akkermansia*, a genus of SCFA-producing bacteria, has received attention as a factor that suppresses high-fat-diet-induced metabolic disorders, including metabolic endotoxaemia and insulin resistance (33). Our results showed *Megamonas* is a more important member as a potential producer of SCFAs, especially in the cecal environment. These results highlight the fact that integrated metagenomic and metatranscriptomic analysis also provides biological interpretations based on two aspects: gene abundance and expression levels.

Finally, we compared the fecal microbiomes of six common marmosets with those of humans and the major model animals macaques, mice, and rats by 16S rRNA gene analysis. The marmoset microbiome was found to be most similar to the human microbiome, with *Bacteroides*, *Bifidobacterium*, and *Collinsella* shared between them. These results suggest marmosets can be expected to be a useful animal model in microbiome studies.

In conclusion, this study developed a method for integrating the metagenome and metatranscriptome for the analysis of multiple intestinal sites. This analysis method allows us to quantify gene expression levels and analyze gene expression changes among intestinal sites, including changes in unknown bacterial genes, which are overlooked by conventional methods. As a result of applying this analysis method to multiple intestinal sites of the common marmoset, we revealed that changes in the internal environment along the intestinal tract may lead to variation in the expression pattern of the microbiome. Moreover, this microbial change may have a mutualistic effect on the environment inside the intestine. These findings highlight the importance of database-independent methods in metatranscriptomic analysis to quantify gene expression in the microbiome.

## MATERIALS AND METHODS

### Animal experiences.

Common marmosets were housed at the Central Institute for Experimental Animals (Kawasaki, Japan) with free access to a pellet diet (for monkeys, CLEA New World Monkey Diet, CMS-1M; CREA Japan, Tokyo, Japan). The cage size was 820 × 610 × 1,578 mm, and the cages were positioned facing each other to allow the animals to communicate visually and vocally. All cages were equipped with a sleeping area, wooden perches, and hammocks. The animal rooms were maintained at 26°C to 28°C and 40% to 60% humidity with a 12 h:12 h light/dark cycle. The animals were negative for Salmonella spp., Shigella spp., and Yersinia pseudotuberculosis in yearly fecal examinations. Two marmosets were selected in this experiment so that the sample volumes from all three sites satisfied the requirements of the experimental protocol. Marmosets were euthanized with intravenous administration of pentobarbital overdose, and the digestive tract was isolated. The gastrointestinal tract of each animal was excised, and the luminal and mucosal content of each gastrointestinal tract site was collected and divided into samples for metagenomic and metatranscriptomic analyses. The contents were immediately frozen in liquid nitrogen and stored at −80°C. The metatranscriptomic analysis samples were crushed and homogenized in solution D containing guanidinium, which inhibits RNase (43), within 1 week after dissection to protect against degradation and stored at −80°C. The luminal and mucosal contents of the cecum and transverse colon and fecal contents of the marmosets (individual ID: I6289M and individual ID: I6027M; Table 10 posted at https://doi.org/10.5281/zenodo.6787048) were used for metagenomic and metatranscriptomic analyses. These three sites were targeted at the beginning, middle, and end of the colon, which has an abundant microbiome. The fecal contents of a total of six marmosets in addition to these two marmosets were used for 16S rRNA gene analysis.

The animal experiment protocol was approved by the CIEA Institutional Animal Care and Use Committee (approval no. 17031). The study was conducted in accordance with the guidelines of CIEA that comply with the Guidelines for Proper Conduct of Animal Experiments published by the Science Council of Japan. Animal care was conducted in accordance with the Guide for the Care and Use of Laboratory Animals (Institute for Laboratory Animal Resources, 2011).

### Shotgun metagenomic sequencing.

DNA was extracted from each metagenomic sample using a MORA-EXTRACT Kit (Kyokuto Pharmaceutical Industrial Co., Ltd., Tokyo, Japan). Sequencing libraries were prepared using the TruSeq Nano DNA Library Prep Kit (Illumina Inc., San Diego, CA, USA). All these procedures were performed according to the kit manufacturer’s instructions (Table 11 posted at https://doi.org/10.5281/zenodo.6787048). Illumina HiSeq sequencing yielded a total of 435 giga nucleotides (Gnt) of paired-end reads (250 nt × 2) for the metagenome. This data set included an average of 145.1 M reads ± 3.9 M reads (mean ± s.d.) per sample before quality filtering, described below, and 125.9 M reads ± 5.3 M reads afterwards (Table 12 posted at https://doi.org/10.5281/zenodo.6787048). Shotgun metagenome libraries were adapter trimmed and quality filtered by Trimmomatic (44) version 0.36 with the following parameters: “ILLUMINACLIP:Adapter.fa:2:30:10:8:true, LEADING:3, TRAILING:3, SLIDINGWINDOW:4:15, MINLEN:50” and FASTX-Toolkit version 0.0.14 with “-q 20 -p 80” (http://hannonlab.cshl.edu/fastx_toolkit/), respectively. Potential host and feed contaminants were then filtered by removing reads with sequences aligned to the host genome and feed genome (Text S1).

### Metatranscriptomic sequencing.

RNA was extracted by a combination of the acid-guanidium-phenol-chloroform RNA extraction method (45) and bead crushing method and assessed to ensure high quality (RNA integrity number [RIN] scores ≥7.9) (Table 13 posted at https://doi.org/10.5281/zenodo.6787048). rRNA was removed using the Ribo-Zero Gold rRNA Removal Kit (Epidemiology) (Illumina). Sequencing libraries were prepared using the TruSeq Stranded Total RNA HT Kit (Illumina). All these procedures were performed according to the manufacturer’s instructions (Table 11 posted at https://doi.org/10.5281/zenodo.6787048). Illumina HiSeq sequencing yielded a total of 165 Gnt of paired-end reads (100 nt × 2) for the metatranscriptome. This data set included an average of 137.7 M reads ± 2.8 M reads (mean ± s.d.) per sample before quality filtering, described below, and 126.7 M reads ± 4.0 M reads afterwards (Table 12 posted at https://doi.org/10.5281/zenodo.6787048). Metatranscriptome libraries were adapter trimmed and quality filtered using the same method as those used for the metagenome libraries. The rRNA reads were removed by SortMeRNA (46) version 2.1 with “-e 1e-30.” Potential host and feed contaminants were filtered in the same way as the metagenome libraries.

### Integrated metagenomic and metatranscriptomic analyses.

The integrated analytical method proposed in this study is composed of three main steps: (i) reconstruction of a common reference metagenome for all sites by assembly, scaffolding and merging (Fig. 1A and B); (ii) mapping of DNA and mRNA reads to the reconstructed reference metagenome (Fig. 1C and D); and (iii) quantification of microbial gene expression at the whole-community and per-cell levels (Fig. 1E). Evaluation of this analytical method and determination of parameters for each step were carried out by using the genomes of known bacterial species (Table 14, 15, and 16 posted at https://doi.org/10.5281/zenodo.6787048; Text S1).

The sequenced DNA reads were assembled by Megahit (47) version 1.1.3 with “-k-min 21, -k-max 141, -k-step 12, -prune-depth 20.” Contigs shorter than 1,000 bp were discarded from further processing. The contigs were scaffolded by OPERA-LG (48) version 2.0.6 using paired-end read information. By merging the scaffolds of metagenomes from three intestinal sites using QuickMerge (49) version 0.3 with “-hco 50, -c 50, -mL 1000,” the common reference metagenomic sequences were reconstructed. These parameters were decided by assessing the accuracy of the genomes constructed at each step. QuickMerge is a tool developed for merging contigs from long-read assembly and hybrid assembly of the same sample. We applied this tool to merge metagenomic contigs between sites. The merging parameters need to be set appropriately to avoid incorrect pairing between overlapping regions since this usage differed from the original design of QuickMerge in two ways: (i) the metagenome contains a variety of bacterial genomes, and hence merging contigs is more complex than merging contigs from a single species; and (ii) the base-calling accuracy of short-read is higher than those of long-read assemblies (50). Therefore, we determined the optimal combination of parameters by evaluating the genome construction accuracy among multiple combinations of parameter values: the alignment confidence “-c,” overlap confidence “-hco,” and merge length cutoff “-mL,” which is higher than the default setting (Text S1). Gene-coding regions were then predicted in the reference metagenomic sequences by MetaGeneMark (51) version 3.38 to generate the entire list of genes in the intestinal sites. We used GhostKOALA (52) and DIAMOND blastp (53) version 0.9.21.122 with “–evalue 1e-10, –query-cover 85” to annotate the predicted genes according to orthologous groups in the KEGG database (release 94.1) and the COG database (21). Subsequently, mRNA reads were mapped to the metagenomic reference sequences by Bowtie2 (54) version 2.3.4.3, and the number of mRNA reads was counted by HTSeq (55) version 0.9.1 to quantify the gene expression level. DNA reads were also mapped to the metagenomic reference sequences by Bowtie2 version 2.3.4.3 with “-x 2000,” and the coverage of each metagenomic sequence was calculated by Samtools (56) version 0.1.19.

### Covariation analysis incorporating bivariate spatial relevance.

We performed covariation analysis to predict the functions of unknown genes. This analysis was based on the assumption that functionally similar genes are covariant in their expression levels (23). First, we benchmarked using the profiles of expression at the whole-community and per-cell levels by assessing the accuracy of this covariation analysis in classifying the known genes with the same metabolic process. We grouped the known genes into gene clusters by COG annotation and calculated the bivariate spatial association measure (L statistic value) (24) to detect covarying gene pairs in a six-dimensional vector of expression levels in three sites in two individuals. This benchmark was used to evaluate the covariation analysis method by AUCs of FPR versus sensitivity along the L statistic value and to determine the threshold of the L statistic value to guarantee FPR < 0.05. As a result of the benchmarking, we found that using the expression levels for the whole community was more accurate than using the expression levels per cell. Next, we grouped the unknown genes into gene clusters by protein sequence similarity using MMSEQS2 (57). We performed covariation analysis on the unknown and known gene clusters together. This allowed us to predict the functions of the unknown gene cluster when the known and unknown gene clusters were linked (Table 9 posted at https://doi.org/10.5281/zenodo.6787048; Text S1).

### Quantification of gene expression levels.

Metatranscriptomic functional activity was assessed with two quantification methods. The first was a general method to quantify gene expression by normalizing mRNA read counts with transcripts per million (TPM) (this is called the “gene expression level in the whole community” in this study). This method can estimate metatranscriptomic activity in a microbial community. The second method was normalization of the mRNA read counts with DNA coverage, thus estimating the gene expression level per single bacterium (this parameter is called the “gene expression level per cell” in this study) (Text S1).

### Taxonomic profiling.

Each reconstructed genome was identified at the taxon level by mapping the predicted genes against the nonredundant protein database and assigning taxonomic annotations with a voting-based approach using CAT version 4.3.3 (58).

### 16S rRNA gene sequencing and comparison among animal models.

To compare the common marmoset fecal microbiomes with those of humans and other major animal models, 16S rRNA sequencing was conducted on fecal samples from 6 marmosets. Marmoset fecal DNA was extracted from each metagenomic sample using the MORA-EXTRACT Kit (Kyokuto Pharmaceutical Industrial Co., Ltd., Tokyo, Japan) by the bead crushing method. The 16S rRNA V3 to V4 region was amplified using the KAPA HiFi HotStart ReadyMix PCR Kit (KAPA BioSystems, USA). For PCR, the forward primer 5′-CCTACGGGNGGCWGCAG-3′ and reverse primer 5′-GACTACHVGGGTATCTAATCC-3′ were used. Sequencing libraries were prepared using the Nextera XT Kit (Illumina) (Table 11 posted at https://doi.org/10.5281/zenodo.6787048). All these procedures were performed according to the kit manufacturers’ instructions. Sequencing was performed using an Illumina MiSeq sequencer, which yielded a total of 11.3 Gnt of paired-end reads (forward: 350 bp, reverse: 250 bp). This data set included an average of 3,154,000 ± 1,190,000 reads per sample before quality filtering and 1,387,000 ± 346,000 reads afterwards (Table 12 posted at https://doi.org/10.5281/zenodo.6787048). The sequences were analyzed using Quantitative Insights into Microbial Ecology (QIIME; version 1.9.1) (59). The 16S rRNA gene sequence data for fecal samples from humans, macaques, rats, and mice were obtained from a previous study (34) (Text S1). To avoid any bias from differences in sequencing depths, the OTU table was rarefied to the lowest number of sequences per sample.

### Comparison of the accuracy of metagenome reconstruction methods.

We compared the metagenome reconstruction method proposed in this study with other existing methods, including simple coassembly. Furthermore, we validated the methods using only metagenomic reads (MG) and using both metagenomic and metatranscriptomic reads (MG+MT) as inputs. The methods were as follows: (i) merging (MG) (our method): briefly restated, the metagenomic reads were assembled per site, and the generated scaffolds were subsequently merged among multiple sites; (ii) merging (MG+MT): same method as (i), but included the metatranscriptomic reads as well as the metagenomic reads to be assembled; (iii) coassembly (MG): the metagenomic reads of all sites were simply coassembled and scaffolded; (iv) coassembly (MG+MT): same method as (iii), but included the metatranscriptomic reads as well as the metagenomic reads to be assembled; (v) MOSCA (60): a pipeline for metatranscriptomic analysis, but because it is not specifically designed to integrate multiple sites as in this study, we used a concatenated metagenomic read file among multiple sites as input for the comparison; (vi) IMP3 (61): a pipeline for hybrid assembly of metagenomic and metatranscriptomic reads, but because it is not specifically designed to integrate multiple sites as in this study, we used a concatenated metagenomic and metatranscriptomic read file among multiple sites as input; (vii) merging (two individuals): the metagenomes reconstructed per individual in (i) were further merged between individuals. In (v) and (vi), the pipeline was executed with default settings. These reconstruction methods were assessed using MetaQUAST (62) by feeding the final contigs output from each method. The data set used as input contained DNA and RNA sequence reads assigned to the top 20 bacterial species in the taxonomic profile by Kraken2 (63) (Table 14 posted at https://doi.org/10.5281/zenodo.6787048). The assessment index to evaluate the assembly performance was the composite performance metric (CPM) proposed for assembler evaluation in a previous study (40). The CPM was computed based on the information derived from the results of MetaQUAST (62) (see Text S1for details on calculation methods).

### Data and source code availability.

All raw sequence data have been submitted to the DDBJ under project PSUB014668 from the Ministry of Education, Culture, Sports, Science, and Technology of Japan. The codes and pipelines used in this study for metagenome assembly, gene expression analysis, and spatial covariation analysis are all available at https://github.com/MikaUhr/IMPIA.git. The gene expression profile and gene annotation profile are available at https://doi.org/10.5281/zenodo.6782852.
