# Supplementary figures and images for: Intraintestinal Analysis of the Functional Activity of Microbiomes and Its Application to the Common Marmoset Intestine
Source: mSystems. 2022 Aug 25;7(5):e00520-22. doi: 10.1128/msystems.00520-22 (PMC9601136; doi:10.1128/msystems.00520-22)

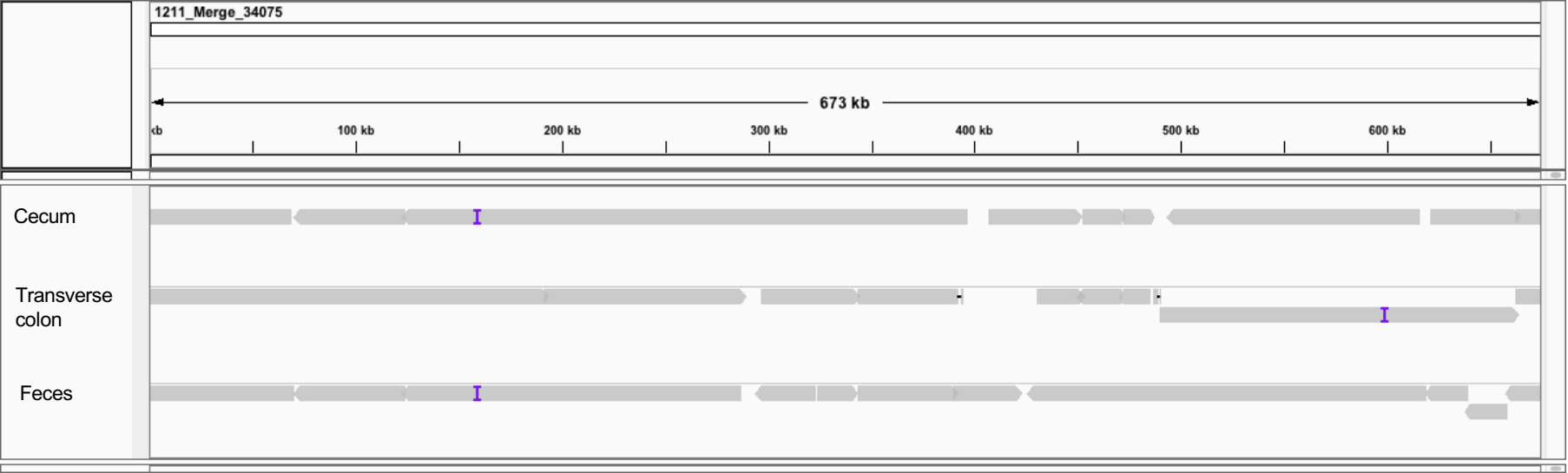

Supplement: FIG S1 [file msystems.00520-22-sf001.pdf]

**Cecum**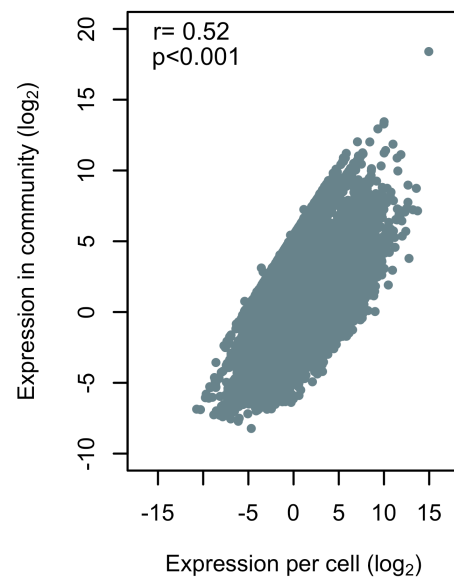**Transverse colon**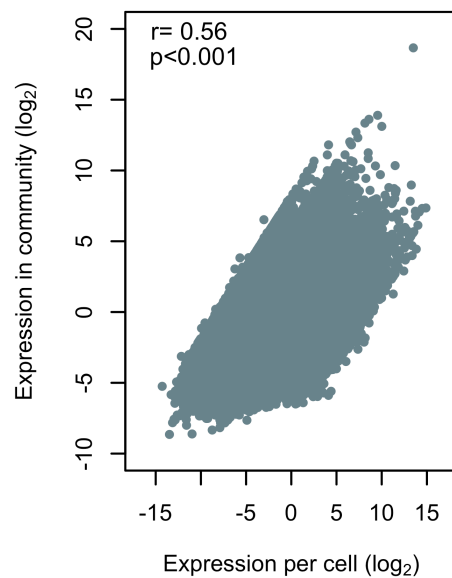**Feces**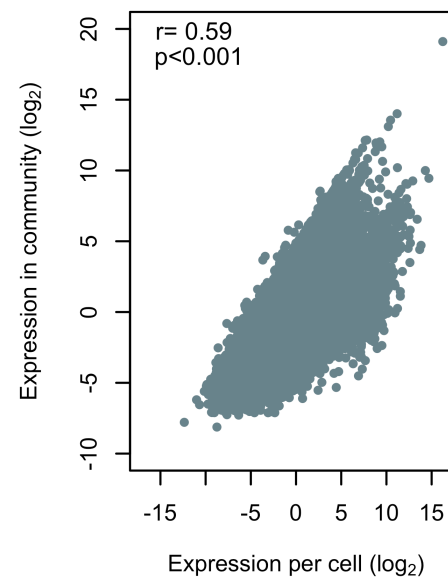**Cecum**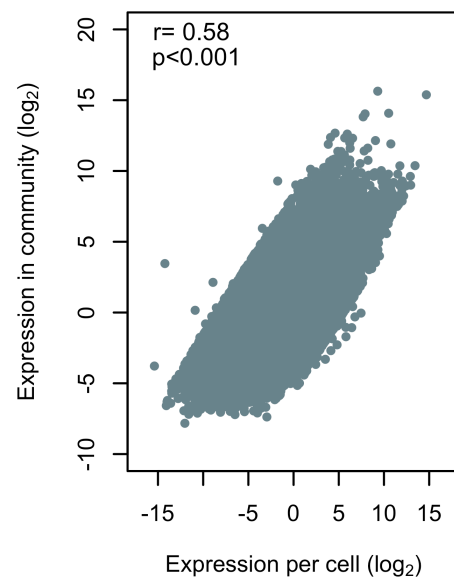**Transverse colon**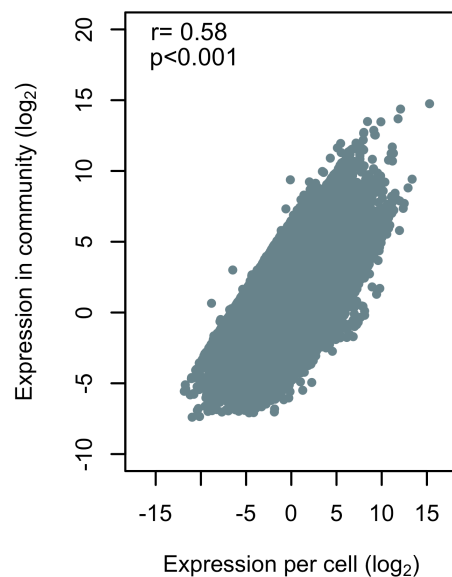**Feces**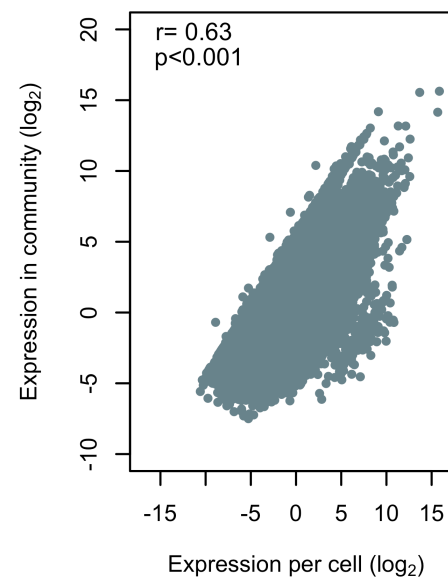

Supplement: FIG S2 [file msystems.00520-22-sf002.pdf]

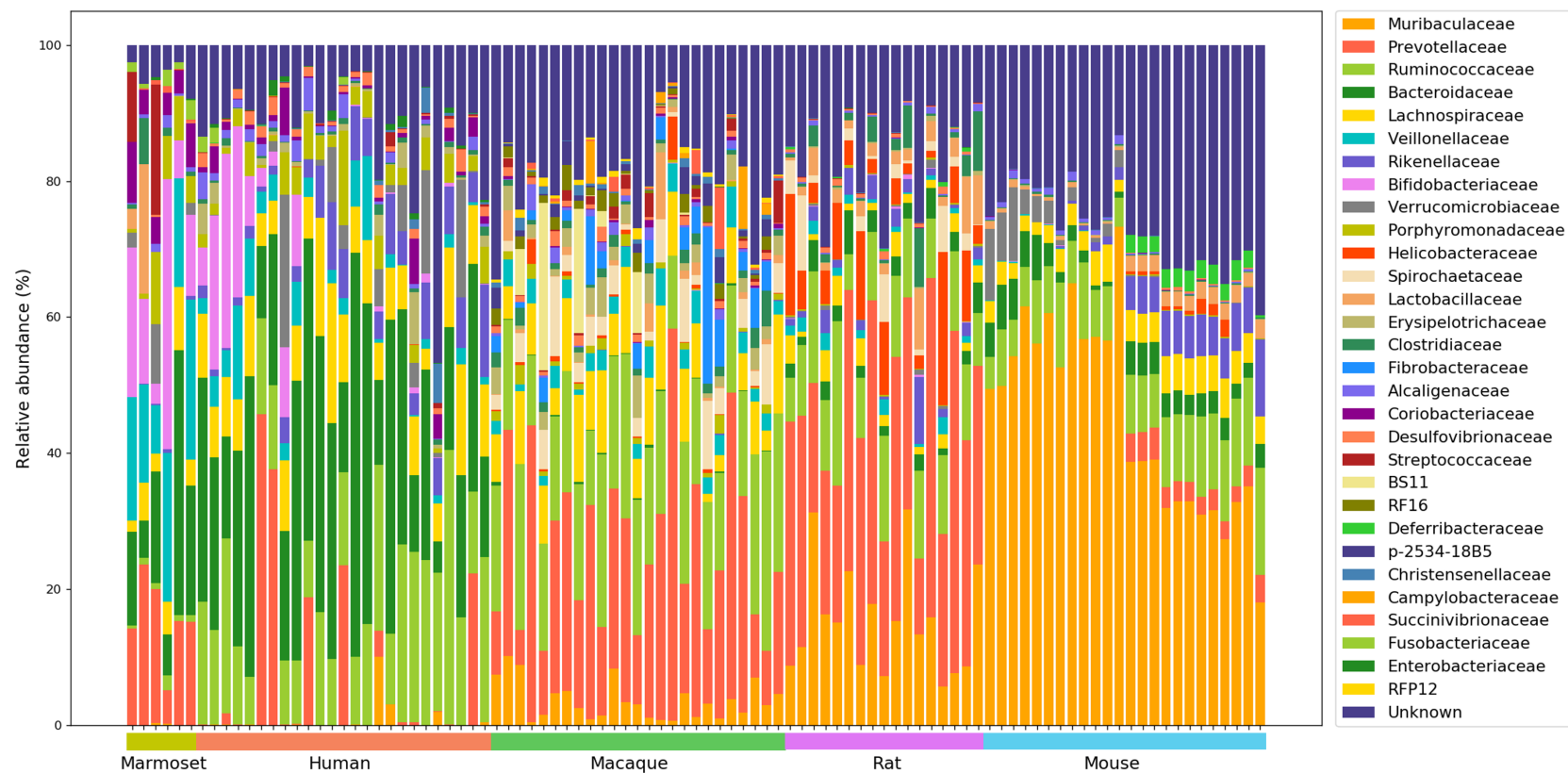

Supplement: FIG S3 [file msystems.00520-22-sf003.pdf]
