# Supplementary material for: Intraintestinal Analysis of the Functional Activity of Microbiomes and Its Application to the Common Marmoset Intestine
Source: mSystems. 2022 Aug 25;7(5):e00520-22. doi: 10.1128/msystems.00520-22 (PMC9601136; doi:10.1128/msystems.00520-22)
Supplement: TABLE S1 [file msystems.00520-22-st001.docx]

Table S1. Number of annotated genes

| **Individual** | **Total number of genes** | **Annotated by COG** | **Annotated by KEGG** |
| --- | --- | --- | --- |
| Individual 1 | 246,980 | 183,649 | 134,191 |
| Individual 2 | 320,613 | 232,038 | 167,769 |
