# Supplementary material for: Intraintestinal Analysis of the Functional Activity of Microbiomes and Its Application to the Common Marmoset Intestine
Source: mSystems. 2022 Aug 25;7(5):e00520-22. doi: 10.1128/msystems.00520-22 (PMC9601136; doi:10.1128/msystems.00520-22)
Supplement: TEXT S1 [file msystems.00520-22-s0001.pdf]

Supplementary Notes

Intra-intestinal analysis of the functional activity of microbiomes and its application to the common marmoset intestine

Mika Uehara[1], Takashi Inoue[2], Minori Kominato[1], Sumitaka Hase[1], Erika Sasaki[2, 4], Atsushi Toyoda[3], Yasubumi Sakakibara[1*]

[1)] Department of Biosciences and Informatics, Keio University, Yokohama, Kanagawa 223-8522, Japan
[2)] Department of Marmoset Biology and Medicine, Central Institute for Experimental Animals, Kawasaki, Kanagawa 210-0821, Japan
[3)] Department of Genomics and Evolutionary Biology, National Institute of Genetics, Mishima, Shizuoka 411-8540, Japan
[4)] Laboratory for Marmoset Neural Architecture, RIKEN Center for Brain Science, 2-1 Hirosawa, Wako-shi, Saitama 351-0198, JAPAN.

*Corresponding Author
Yasubumi Sakakibara
3-14-1 Hiyoshi, Kohoku-ku, Yokohama, 223-8522, Japan
Phone/Fax: +81-45-566-1791
E-mail: yasu@bio.keio.ac.jp

Section 1. Visualization of scaffold alignment to the merged genome by IGV
We reconstructed the metagenomes by merging scaffolds among sites. To visualize the scaffolds that construct the merged genome, the scaffolds at each site were aligned with the merged genome by minimap2 version 2.17-r941 with "-g 100 -r 100 --no-long-join". A BAM file was loaded to IGV (1) version 2.7.2. The scaffolds across the sites complemented each other to reconstruct a large genome, and the merging improved the contiguity of the genome (Fig. S1).

Section 2. Percentage of genes that match in scaffolds of all three sites and the reconstructed scaffold
We verified that the gene annotations were retained before and after merging by examining the percentage of genes common to the three sites that matched the corresponding genes in the reconstructed metagenome. First, we aligned each site scaffold to the reconstructed metagenome using minimap2 version 2.17-r941 (2) with "-g 100 -r 100 --no-long-join" for the gene position adjustment. Specifically, we converted the positions of the gene regions of the scaffolds at each site to the positions on the reconstructed metagenome based on the PAF file with cs tags from the alignment result. We then extracted only the genes common to all three scaffolds, excluding genes on scaffolds constructed from one or two sites, to avoid overestimating the percentage of gene matches before and after the merge. The mismatches (including insert, deletion, variant) of gene regions between the scaffold from three sites and the reconstructed metagenome were calculated for known genes. Genes with a maximum mismatch ≤3 bases in the three sites were identified as the genes common to the three sites (Table S3).

Section 3. Functional annotation for unknown genes by covariation analysis
We performed a covariation analysis by modifying some of the methods of previous studies (3) to estimate the function of unknown genes that have no detectable homology with known sequences (Supplementary Note Section 4). This analysis is based on the assumption that functionally similar genes are covariant in their expression levels. As shown below, we first benchmarked the expression profiles of the known genes and then estimated the function of the unknown genes using the models determined in this benchmark.

Benchmarking with known genes: Benchmarking was performed to determine and evaluate the model that most accurately discriminates genes with common metabolic processes. Known genes are genes annotated by the COG database (4) and the KEGG database (5), as described in "Integrated metagenomic and metatranscriptomic analyses" in this paper. We grouped the genes with the same COG ID into known gene clusters and summed the expression levels within each known gene cluster. We excluded gene clusters with an expression variance of 1.00 or less among the three sites. The metabolic process of each known gene cluster was applied to the metabolic process of the gene with the longest sequence in the cluster as a representative. In order to relate the genes between individuals, we used the known gene clusters with COG IDs present in both individuals 1 and 2 for covariation analysis. After these pre-processing steps, we calculated the bivariate spatial association measure (L statistic) (6) of expression levels between all known gene pairs in a six-dimensional vector consisting of three sites in two individuals. L statistic for gene expression levels X and Y was calculated by:

$$L_{X,Y} = \frac{\sum_i \left[\left(\sum_j w_{ij} (x_j - \bar{x}) \right) \cdot \left(\sum_j w_{ij} (y_j - \bar{y}) \right) \right]}{\sqrt{\sum_i (x_i - \bar{x})^2} \sqrt{\sum_i (y_i - \bar{y})^2}}$$

where w_{ij} is a row-standardized version of a spatial wight v_{ij}, which is defined as:

$$v_{ij} = \begin{cases} {d_{ij}}^{-b} & (i \neq j) \\ 1 & (i = j) \end{cases}$$

where d_{ij} refers to the distance between site i and site j (Table S17), and b to the distance friction coefficient ($b = 2$).

By applying L to covariate analysis, we calculated the covariation values between two gene expression levels, taking into account the spatial information of the intestinal sites; for example, the distance between the cecum and transverse colon is closer than the distance between the cecum and anus (feces). The known gene cluster pair was defined as a covariate gene if L is greater than a threshold value. True positives (TPs) were defined as pairs of covariant genes with a common metabolic process definition. False positives (FPs) were defined as pairs of covariant genes without common metabolic linkage definition. True negatives (TNs) were defined as pairs of non-covariant genes without a common metabolic process. False negatives (FNs) were defined as pairs of non-covariant genes with a common metabolic process. The ROC curve was plotted by calculating the false positive rate (FPR; $\mathrm{FPR} = \mathrm{FP} / (\mathrm{FP} + \mathrm{TN})$) and sensitivity ($sensitivity = \mathrm{TP} / (\mathrm{TP} + \mathrm{FN})$) while varying the threshold of the L from 0.00 to 1.00 in 0.01 increments. We assessed the prediction accuracy by calculating the AUC (Fig. 3 A, B and C). As a result of this benchmarking, the model with the highest accuracy (using gene expression profiles at the whole community and KEGG reaction as the metabolic linkage definition) was used for subsequent covariation analysis to estimate the function of unknown genes (Table S8). Threshold of 0.885 was applied as a value to guarantee FPR<0.05.

Estimation of unknown gene functions: We grouped unknown genes by protein sequence similarity using MMSEQS2 (7) with "–cov-mode 1, –cluster-mode 2, -c 0.9, -s 7, –kmer-per-seq 20" as unknown gene clusters and summed the expression levels within each unknown gene cluster. As in the benchmark pre-processing, we excluded gene clusters with an expression variance of 1.00 or less among the three sites and used gene clusters present in both individuals 1 and 2 for covariation analysis. Using the model determined by the benchmark, the unknown and known gene clusters were combined for the covariation analysis. We estimated the metabolic process of the known gene cluster as that of the unknown gene cluster when a known gene cluster was linked to an unknown gene cluster. We identified 3,528 unknown gene clusters that are significantly linked to known gene clusters (Table S6). To find potential functional trends of unknown gene clusters, we examined the functional categories that shared by a large number of gene clusters by enrichment analysis with Fisher's exact test. A functional category was considered significantly different between the known gene clusters and unknown gene clusters if the Benjamini-Hochberg adjusted P value was <0.01 (Fig. 3D).

Section 4. Validation of covariation analysis by sequence similarity of linked gene clusters

In the covariation analysis, we estimated the function of unknown genes by linking gene clusters using the variation of gene expression levels. We validated that the functions estimated by this covariation analysis were functions that could not be annotated by sequence similarity. Therefore, we identified linked gene cluster pairs with sequences that are even remotely similar using DIAMOND blastp version 0.9.21.122 (8) with "--evalue 0.1", and found that no sequences were similar between the clusters. This result shows that covariation analysis using gene expression levels links a potential function that cannot be annotated by sequence similarity.

Section 5. Assessment of reconstructed metagenomics

We evaluated the integrated analytical method using the dataset constructed from top 20 bacterial species in the taxonomic profile by Kraken2 (9) (Table S14). The DNA reads from the dataset were reconstructed through the assembly, scaffolding and merging steps. The reconstructed genomes were assessed by the non-chimera rate, which is the ratio of the length of all chimeric sequences to the total length of reconstructed genomes. A non-chimera rate = 1 indicated that the genome was reconstructed with high accuracy without chimeras. The chimeric sequences refer to a mixture of genomes from multiple species, which was calculated by aligning the sequences of 20 species in the database to the reconstructed genome using Blastn with the following threshold: "evalue<1e-10, alignment length≥500 bp, identity≥85%".

Section 6. Removal of contaminated sequences

Potential host and feed contaminants were then filtered by removing reads with sequences aligned to the host genome and feed genome. The sequenced reads were aligned into the common marmoset (Callithrix jacchus; GenBank assembly accession GCA_000004665.1), bread wheat (Triticum aestivum; GenBank assembly accession GCA_900519105.1), soybean (Glycine max; GenBank assembly accession GCA_000004515.4) and atlantic cod (Gadus morhua; GenBank assembly accession GCA_902167405.1) genomes by Bowtie2 version 2.3.4.3 (10) with "-x 2000" for DNA and HISAT2 version 2.1.0 (11) for RNA reads, respectively.

Section 7. Computation method of gene expression level per cell

The functional activity of the microbiome was assessed with the gene expression level at both the whole community and per cell levels. The gene expression levels at the whole community were normalized to corresponding gene abundance to obtain an estimate of gene expression levels per cell T as follows:

$$T_{g,s,i} = \begin{cases} \frac{R_{g,i}}{\sum_g R_{g,i}} \times \frac{\sum_{s \ni g} D_{s \ni g,i}}{D_{s \ni g,i}}, & if\ D_{s \ni g,i} > 0 \\ 0, & if\ D_{s \ni g,i} = 0 \end{cases}$$

$R_{g,i}$ is the count of mRNA reads in TPM of gene g in sample i. $D_{s \ni g,i}$ is the count of DNA reads in TPM of the genome sequences where the gene g is located within sample i.

Section 8. Parameter determination and evaluation of the integrated analytical method

The three steps of the integrated analytical method to reconstruct the metagenome—assembly, scaffolding and merging—were assessed using real DNA reads assigned to the top 20 bacterial species in the taxonomic profile by Kraken2 (10) as input (Table S14). For DNA assembly, Megahit (12) was run with all combinations of parameters, as follow: --k-min 35; --k-max 225; --k-step 12; and --prune-depth 2, 5, 10 or 20. For reference metagenomic sequence reconstruction, QuickMerge (13) was run with the following parameters: -hco 5, 10, 50 or 100; -c 1.5, 10, 50 or 100; and -ml 1,000, 5,000 or 10,000.

To assess the accuracy of the genomes constructed at each step, the number of genes covered for each non-chimeric reconstructed genome were used as criteria. The reconstructed genome was aligned to in the database containing the genomes of the top 20 bacterial species by Blastn (14) with the following threshold: "evalue<1e-10, alignment length≥500 bp, identity≥85%", and one reconstructed genome aligned to multiple bacterial genomes was defined as a chimera. The covered genes represent the number of perfectly matched genes upon alignment of the genes

against the reconstructed genome. For only the merging step, the number of genes was counted as 1/N if the same gene appeared multiple times (N), which can determine the parameters that were merged correctly. We detected parameter at the assembly and merge steps by assessing the number of genes covered by non-chimera genome (Table S15 and S16).

Section 9. Information of the data on animals other than marmosets used for 16S rRNA analysis

The 16S rRNA gene sequence data for fecal samples from humans, macaques, rats, and mice were obtained from a previous study (15) and the following animal information is from descriptions of this previous study. The data include in-bred C57BL/6 mice (male; n=24; mean age 14 weeks ;diet: normal chow ad libitum); outbred NIH heterogeneous stock rats (16, 17) (male; n = 17; 18 weeks of age after 12 weeks on a low fat or high fat diet: 8 rats were on a high fat diet (60% kcal from fat) and 9 rats were on a low fat diet (10%kcal from fat) ad libitum); healthy adult NHPs (cynomolgus macaques; female; n = 25; mean age: 8.8 years; maintained on a human-style Mediterranean or Western diet for the last 34 month (18); and human subjects (n = 25; female/male 18/7; mean age: 39.3 years). Rat diets were purchased from Research Diets (LF: D12492; HF: D12450J). All mice and rats were maintained at the Wake Forest Biotech Place Animal Resource Program facility, and the NHPs were maintained at the Wake Forest University Primate Center. The western diet consisted of lard, beef tallow, butter, egg, cholesterol, casein, lactalbumin, dextrin, high-fructose corn syrup, and sucrose; while the Mediterranean diet comprised fish oil, olive oil, fish meal, butter, egg, black and garbanzo bean flour, wheat flour, V-8 juice, fruit puree, and sucrose.

Section 10. Evaluation metrics for comparing the metagenomic reconstruction methods

The composite performance metric (CPM) (19) used to compare the reconstruction methods was computed based on the chimera index (CI), max alignment length (MAL) and total alignment length (TAL) derived from the results of MetaQUAST (20). Specifically, for a given M: total length of all regions aligned to the reference genomes and N: total length of all regions unaligned to the reference genomes, the definition of these metrics proposed in a previous study (19) are as follows:

$CI\ (\%) = N/\ (M + N) \times 100\%$

MAL: max(all regions aligned to the reference genomes in the full set of contigs)

TAL: total of all regions aligned to the reference genomes in contigs that are 1000 bp or greater by merging the overlapped regions

All metrics was normalized from 0-5 as in the previous study (19), with larger values indicating better performance. The CI_{NR}, MAL_{NR} and TAL_{NR}, which is normalized each metric were calculated as:

$$CI_{NR} = 5 \times (1 - CI/max(CI\ of\ all\ methods)).$$

Similarly, other metrics was defined:

$$MAL_{NR} = 5 \times max(MAL\ of\ all\ methods),$$

$$TAL_{NR} = 5 \times max(TAL\ of\ all\ methods).$$

The CPM is the weighted average of above metrics about contig qualities:

$$CPM = 0.5 \times CI_{NR} + 0.25 \times (MAL_{NR} + TAL_{NR}).$$

We determined the best performing reconstruction method by computing the CPM.

Reference

1. Thorvaldsdóttir H, Robinson JT, Mesirov JP. 2013. Integrative genomics viewer (IGV): high-performance genomics data visualization and exploration. Brief Bioinform 14:178–192.
2. Li H. 2018. Minimap2: pairwise alignment for nucleotide sequences. Bioinformatics;34:3094-100.a
3. Salazar G, Paoli L, Alberti A, Huerta-Cepas J, Ruscheweyh HJ, Cuenca M, Field CM, Coelho LP, Cruaud C, Engelen S, Gregory AC. 2019. Gene expression changes and community turnover differentially shape the global ocean metatranscriptome. Cell 179:1068-1083.
4. Galperin MY, Makarova KS, Wolf YI, Koonin E V. 2015. Expanded Microbial genome coverage and improved protein family annotation in the COG database. Nucleic Acids Res 43:D261–D269.
5. Kanehisa M, Goto S. 2000. KEGG: kyoto encyclopedia of genes and genomes. Nucleic Acids Res 28:27–30.
6. Lee SI. 2001. Developing a bivariate spatial association measure: An integration of Pearson's r and Moran's I. J Geogr Syst 3:369–385.
7. Steinegger M, Söding J. 2017. MMseqs2 enables sensitive protein sequence searching for the analysis of massive data sets. Nat Biotechnol 35:1026–1028.
8. Buchfink B, Xie C, Huson DH. 2015. Fast and sensitive protein alignment using DIAMOND. Nat Methods 12:59–60.
9. Wood DE, Lu J, Langmead B. 2019. Improved metagenomic analysis with Kraken 2. Genome Biol 20:1-13.
10. Langmead B, Salzberg SL. 2012. Fast gapped-read alignment with Bowtie 2. Nat Methods 9:357–359.
11. Kim D, Langmead B, Salzberg SL. 2015. HISAT: A fast spliced aligner with low memory requirements. Nat Methods12:357–360.
12. Li D, Liu CM, Luo R, Sadakane K, Lam TW. 2015. MEGAHIT: An ultra-fast single-node solution for large and complex metagenomics assembly via succinct de Bruijn graph. Bioinformatics 31:1674–1676.
13. Chakraborty M, Baldwin-Brown JG, Long AD, Emerson JJ. 2016. Contiguous and accurate de novo assembly of metazoan genomes with modest long read coverage. Nucleic Acids Res 44:e147-e147.
14. McGinnis S, Madden TL. 2004. BLAST: at the core of a powerful and diverse set of sequence analysis tools. Nucleic Acids Res 32:W20–25.
15. Nagpal R, Wang S, Solberg Woods LC, Seshie O, Chung ST, Shively CA, Register TC, Craft S, McClain DA, Yadav H. 2018. Comparative microbiome signatures and short-chain fatty acids in mouse, rat, non-human primate, and human feces. Front Microbiol 9: 2897. https://doi.org/10.3389/fmicb.2018.02897
16. Hansen C, Spuhler K. 1984. Development of the National Institutes of Health genetically heterogeneous rat stock. Alcoholism: Clinical and Experimental Research 8:477-9.
17. Woods LC, Mott R. 2017. Heterogeneous stock populations for analysis of complex traits, p 31-44. InSystems Genetics Humana Press, New York, NY.

18. Nagpal R, Shively CA, Appt SA, Register TC, Michalson KT, Vitolins MZ, Yadav H. 2018. Gut microbiome composition in non-human primates consuming a Western or Mediterranean diet. Front Nutr 25;5:28.
19. Deng X, Naccache SN, Ng T, Federman S, Li L, Chiu CY, Delwart EL. 2015. An ensemble strategy that significantly improves de novo assembly of microbial genomes from metagenomic next-generation sequencing data. Nucleic Acids Res 43:e46-e46.
20. Mikheenko A, Saveliev V, Gurevich A. 2016. MetaQUAST: evaluation of metagenome assemblies. Bioinformatics 32:1088-90.
