# Supplementary material for: Intraintestinal Analysis of the Functional Activity of Microbiomes and Its Application to the Common Marmoset Intestine
Source: mSystems. 2022 Aug 25;7(5):e00520-22. doi: 10.1128/msystems.00520-22 (PMC9601136; doi:10.1128/msystems.00520-22)
Supplement: TABLE S2 [file msystems.00520-22-st002.docx]

Table S2. Number of expressed genes at each site

| **Individual** | **Site** | **Number of predicted genes** | **Number of expressed genes** | **Percentage of expressed genes** |
| --- | --- | --- | --- | --- |
| Individual 1 | Cecum | 246,980 | 56,550 | 22.9% |
|  | Transverse colon |  | 89,131 | 36.1% |
|  | Feces |  | 56,552 | 22.9% |
|  | At any of 3 sites |  | 100,174 | 40.6% |
|  | At all 3 sites |  | 36,825 | 14.9% |
| Individual 2 | Cecum | 320,613 | 97,523 | 30.4% |
|  | Transverse colon |  | 94,065 | 29.3% |
|  | Feces |  | 103,432 | 32.3% |
|  | At any of 3 sites |  | 140,707 | 43.9% |
|  | At all 3 sites |  | 60,594 | 18.9% |
