# Supplementary material for: Intraintestinal Analysis of the Functional Activity of Microbiomes and Its Application to the Common Marmoset Intestine
Source: mSystems. 2022 Aug 25;7(5):e00520-22. doi: 10.1128/msystems.00520-22 (PMC9601136; doi:10.1128/msystems.00520-22)
Supplement: TABLE S3 [file msystems.00520-22-st003.docx]

Table S3. Percentage of genes that match in scaffolds of all 3 sites and reconstructed scaffolds

| **Acceptable mismatch** | **Individual 1** | **Individual 2** |
| --- | --- | --- |
| ≤1bp | 94.1% | 92.1% |
| ≤3bp | 96.1% | 95.0% |
| ≤5bp | 97.0% | 96.3% |
| ≤10bp | 97.9% | 97.7% |
