# Supplementary material for: Intraintestinal Analysis of the Functional Activity of Microbiomes and Its Application to the Common Marmoset Intestine
Source: mSystems. 2022 Aug 25;7(5):e00520-22. doi: 10.1128/msystems.00520-22 (PMC9601136; doi:10.1128/msystems.00520-22)
Supplement: TABLE S4 [file msystems.00520-22-st004.docx]

Table S4. N50, composite performance metric (CPM), chimera index (CI), max alignment length (MAL), and total alignment length (TAL) of the metagenome reconstructed by each method

| **Evaluation index** | | **CPM** | | **CI** | | **MAL (bp)** | | **TAL (bp)** | | **N50 (bp)** | |
| --- | --- | --- | --- | --- | --- | --- | --- | --- | --- | --- | --- |
| **Individual** | | **I1** | **I2** | **I1** | **I2** | **I1** | **I2** | **I1** | **I2** | **I1** | **I2** |
| 1 | Merging (MG) | **2.91** | **3.48** | **11.5%** | **6.0%** | **250,951** | 672,143 | 15,455,965 | 15,815,576 | **48,003** | **26,119** |
| 2 | Merging (MG+MT) | 2.79 | 3.27 | 11.9% | 6.2% | **250,951** | 608,158 | 14,689,440 | 15,202,849 | 42,065 | 16,723 |
| 3 | Co-assembly (MG) | 2.74 | 2.56 | 11.8% | 6.8% | 219,038 | 289,499 | 15,886,459 | 15,554,461 | 32,925 | 14,940 |
| 4 | Co-assembly (MG+MT) | 2.64 | 3.03 | 12.3% | 7.4% | 210,357 | 608,158 | 16,221,863 | 15,871,595 | 30,023 | 15,398 |
| 5 | MOSCA | 2.49 | 2.50 | 14.9% | 10.5% | 250,950 | **672,365** | 17,347,569 | **16,904,167** | 27,775 | 20,957 |
| 6 | IMP3 | 2.53 | 2.50 | 14.6% | 10.0% | **250,951** | 608,150 | **17,529,958** | 16,885,627 | 24,717 | 19,990 |
