# Supplementary material for: Intraintestinal Analysis of the Functional Activity of Microbiomes and Its Application to the Common Marmoset Intestine
Source: mSystems. 2022 Aug 25;7(5):e00520-22. doi: 10.1128/msystems.00520-22 (PMC9601136; doi:10.1128/msystems.00520-22)
Supplement: TABLE S5 [file msystems.00520-22-st005.docx]

Table S5. Average of CI and N50 of two individuals in Table S4 (1-6) and those of the metagenome merging the two individuals (7)

| **Evaluation index** | | **CI** | **N50 (bp)** |
| --- | --- | --- | --- |
| 1 | Merging (MG)  (our method) | **8.8%** | **37,061** |
| 2 | Merging (MG+MT) | 9.1% | 29,394 |
| 3 | Co-assembly  (MG) | 9.4% | 23,933 |
| 4 | Co-assembly (MG+MT) | 10.0% | 22,711 |
| 5 | MOSCA | 12.7% | 24,366 |
| 6 | IMP3 | 12.3% | 22,354 |
| 7 | Merging (two individuals) | 12.3% | 34,362 |

The results are shown as the average of two individuals.
