# Supplementary material for: Intraintestinal Analysis of the Functional Activity of Microbiomes and Its Application to the Common Marmoset Intestine
Source: mSystems. 2022 Aug 25;7(5):e00520-22. doi: 10.1128/msystems.00520-22 (PMC9601136; doi:10.1128/msystems.00520-22)
Supplement: TABLE S6 [file msystems.00520-22-st006.docx]

Table S6. CI and N50 of metagenome of each site and merged metagenome among three sites

| **Evaluation index** | **CI** | | **N50 (bp)** | |
| --- | --- | --- | --- | --- |
| **Individual** | **I1** | **I2** | **I1** | **I2** |
| Merging (MG) (our method) | **11.5%** | **6.0%** | **48,003** | **26,119** |
| Each-site (Average of three sites) | 11.8% | 6.1% | 36,405 | 9,541 |
| — Each-site (Cecum) | 11.2% | 6.2% | 46,222 | 9,480 |
| — Each-site (T. colon) | 11.8% | 6.0% | 22,709 | 10,616 |
| — Each-site (Feces) | 12.2% | 6.0% | 40,285 | 8,527 |
